# Integrative population pharmacokinetic/pharmacodynamic analysis of nemonoxacin capsule in Chinese patients with community-acquired pneumonia

**DOI:** 10.3389/fphar.2023.912962

**Published:** 2023-02-28

**Authors:** Yuancheng Chen, Xiaojie Wu, Chengyuan Tsai, Liwen Chang, Jicheng Yu, Guoying Cao, Beining Guo, Yaoguo Shi, Demei Zhu, Fupin Hu, Jinyi Yuan, Yang Liu, Xu Zhao, Yingyuan Zhang, Jufang Wu, Jing Zhang

**Affiliations:** ^1^ Institute of Antibiotics, Huashan Hospital, Fudan University, Shanghai, China; ^2^ Key Laboratory of Clinical Pharmacology of Antibiotics, National Health Commission, Shanghai, China; ^3^ National Clinical Research Center for Aging and Medicine, Huashan Hospital, Fudan University, Shanghai, China; ^4^ Phase I Unit, Huashan Hospital, Fudan University, Shanghai, China; ^5^ TaiGen Biopharmaceuticals Beijing Co., Ltd., Beijing, China

**Keywords:** nemonoxacin, community-acquired pneumonia, population pharmacokinetics, pharmacokinetic/pharmacodynamic analysis, *Streptococcus pneumoniae*, *Klebsiella pneumoniae*, Haemophilus, *Staphylococcus aureus*

## Abstract

**Introduction:** Nemonoxacin is an innovative quinolone antibiotic for treatment of community-acquired pneumonia (CAP). As more data are available from clinical studies, it is necessary to perform an integrative pharmacokinetic/pharmacodynamic (PK/PD) analysis to support and justify the optimal dosing regimen of nemonoxacin in clinical practice.

**Methods and Results:** We developed a population PK model using non-linear mixed effect model based on the data of 195 Chinese subjects receiving nemonoxacin in phase I to III clinical trials. The base model was a standard two-compartment PK model defined by clearance (12 L/h) and central volume of distribution (86 L). Covariates included creatinine clearance (CL_cr_), body weight (BW), sex, disease status and food. Compared to the subject with BW 60 kg, C_max_ and ${AUC}_{0‐24,\;\mathrm{ss}}$ reduced by 24% and 19% in the subject with BW 80 kg, respectively. Compared to the subject with CL_cr_ 150 ml/min, ${AUC}_{0‐24,\;\mathrm{ss}}$ and T_1/2_ increased by 28% and 24%, respectively in the subject with CL_cr_ 30 ml/min. Compared to the fasted status, T_max_ of nemonoxacin increased by 1.2 h in the subject with fed status. Effects of sex and disease status on PK parameters were small (change of PK parameters ≤19%). AUC_0–24_/MIC and %T > MIC were identified as the optimal PK/PD indices for predicting clinical efficacy. The AUC_0-24_/MIC target was 63.3, 97.8, and 115.7 against *Streptococcus pneumoniae*, *Staphylococcus aureus*, and *Haemophilus influenzae*, respectively. The %T > MIC target was 7.96% against *Klebsiella pneumoniae*. Monte Carlo simulation showed that treatment with nemonoxacin 500 mg q24 h could attain a PK/PD cutoff value higher than the MIC_90_ against *S. pneumoniae* and *S. aureus*. The corresponding cumulative fraction of response (CFR) was greater than 93%, while nemonoxacin 750 mg q24 h would provide higher PK/PD cutoff value against *Haemophilus parainfluenzae*, and higher CFR (83%) than 500 mg q24 h.

**Conclusion:** Integrative PK/PD analysis justifies the reliable clinical and microbiological efficacy of nemonoxacin 500 mg q24 h in treating CAP caused by *S. pneumoniae*, *S. aureus*, and *K. pneumoniae*, irrespective of patient sex, mild renal impairment, empty stomach or not. However, nemonoxacin 750 mg q24 h would provide better efficacy than 500 mg q24 h for the CAP caused by *H. parainfluenzae* in terms of CFR.

## Introduction

Nemonoxacin is a novel non-fluorinated quinolone developed by TaiGen Biotechnology. It has been approved for treatment of community-acquired pneumonia (CAP) in adult patients. Nemonoxacin has potent *in vitro* activities against target pathogens of CAP. Especially for penicillin-resistant *Streptococcus pneumoniae* and methicillin-resistant *Staphylococcus aureus* (MRSA), nemonoxacin is more active than levofloxacin (Li et al., 2010). In addition, nemonoxacin also has *in vitro* activity against Gram-negative bacteria equivalent to levofloxacin (Arjona et al., 2009; Chen Y H et al., 2009). The clinical study conducted in the United States (US) indicated that nemonoxacin had an apparent linear pharmacokinetic (PK) profile. Early clinical trials have proved the good clinical efficacy of nemonoxacin 500 mg q24 h in CAP patients, which was equivalent to levofloxacin (Chung et al., 2010; Lin et al., 2010; van Rensburg et al., 2010). However, the PK profile of nemonoxacin has not been characterized in CAP patients so far.

CAP is one of the most common infectious diseases threatening human health (Nair & Niederman, 2011), and resulting in heavy medical cost, about 8.4–9.7 billion dollars in the US each year (Arnold et al., 2003). CAP is pulmonary infection caused by the pathogens in community, most of which are *S. pneumoniae* (15%–76%), *H. influenzae* (3%–46%), *S. aureus* (3%–14%), other Gram-negative bacteria (6%–20%), and atypical pathogen such as *Mycoplasma pneumoniae* and *Chlamydia pneumoniae* (Reimer & Carroll, 1998). Quinolones are broad-spectrum antimicrobial agents with potent activity against the pathogens of CAP. Chinese guidelines recommend quinolones as an important treatment of CAP (Respiratory Branch of Chinese Medical Association, 2006). However, quinolone-resistant strains are increasingly prevalent in *S. pneumoniae* and MRSA due to selective pressure caused by extensive and intensive clinical use of quinolone drugs (Rice, 2006). It is therefore urgently needed to develop more innovative quinolone drugs with more potent antibacterial activities and better safety profile. Nemonoxacin is one of the first achievements in such efforts (Wiles et al., 2010).

Pharmacokinetic/pharmacodynamic (PK/PD) analysis aims to develop a mathematical model which correlates PK with PD by comprehensively analyzing the relationship between time, drug concentration, and treatment effect (Gabrielsson & Green, 2009). After incorporating the variability between and/or within individuals, the PK/PD model could show the probability of concentration or response in the specified interval, thus the population PK/PD model could be also regarded as a statistical model. Population PK/PD study is especially useful for simulating the potential results of clinical trial with the available PK and PD data (Williams & Ette, 2000; Lu, 2004). The simulation based on population PK/PD model could improve the decision-making of clinicians in clinical treatment and facilitate the research and development of new drugs. Population PK/PD study has been successfully used in the clinical development of quinolone drugs such as moxifloxacin (Simon et al., 2003), levofloxacin (Preston et al., 1998), and garenoxacin (Van Wart et al., 2004). To our knowledge, no population PK/PD study has been published for nemonoxacin in CAP patients.

Nemonoxacin capsule has been licensed as innovative new drug in China for treatment of CAP. As more and more data are available from phase I to III clinical trials of nemonoxacin capsules (Guo et al., 2012a; Liu et al., 2017; Yuan et al., 2019), it is necessary to perform an integrative population PK/PD study to characterize the time profile of nemonoxacin in CAP patients, identify the potential significant covariates, derive PK/PD targets using logistic regression and other methods, and so to support and justify the optimal dosing regimen of nemonoxacin *via* Monte Carlo simulation.

## Materials and methods

### Data source

The data included in this PK/PD analysis were derived from clinical studies of nemonoxacin capsules, including phase I clinical trial of oral nemonoxacin capsule (Study TG-873870-C-1), phase II clinical trial of oral nemonoxacin capsule in treatment of CAP (Study TG-873870-C-3), and phase III clinical trial to evaluate the efficacy and safety of oral nemonoxacin capsule *versus* levofloxacin in treatment of CAP (Study TG-873870-C-4) (Supplementary Table S1).

All the clinical trials were approved by the Ethics Committee of corresponding study center. The studies were conducted in compliance with good clinical practice guidelines and the Declaration of Helsinki. All subjects signed informed consent before study entry. Nemonoxacin was provided as 250 mg capsules (product of Huayu Wuxi Pharmaceutical Co., Ltd.). The inclusion and exclusion criteria for healthy volunteers and CAP patients were reported previously (Guo et al., 2012a; Liu et al., 2017; Yuan et al., 2019) (Supplementary Material, the first item, page 1–2).

### Assay of nemonoxacin in plasma

Blood sample (4 ml) was collected at each sampling time point, and processed by centrifugation at 4°C (3,000 r/min, 10 min) within 1 h to separate plasma. The plasma (2 ml) was stored at 20°C and sent to the central laboratory at Huashan Hospital within 1 month for determining nemonoxacin concentration using liquid chromatography-tandem mass spectrometry. The assay method was validated successfully (Guo et al., 2012b), evidenced by lower limit of quantification (LLOQ) 0.005 mg/L, and linear range from 0.005 to 1 mg/L (*R* ^2^ > 0.99). The intra- and inter-day precision was 2.2%–5.9% and 2.7%–6.9%, respectively, and intra- and inter-day accuracy was -0.7%–3.8% and −0.5%–11.0%, respectively.

### Efficacy evaluation

The efficacy of nemonoxacin in treatment of CAP, including clinical efficacy and microbiological efficacy, was appropriately evaluated based on the predefined criteria (Supplementary Material).

### PPK analysis

#### Data processing

The inappropriate data were removed in order to improve the robustness of PPK model, such as the data in case of poor subject compliance, mishandling of blood samples, inappropriately higher plasma concentrations or outliers, plasma concentrations lower than LLOQ, significant deviation of plasma concentration at the same time point based on Chauvenet Criterion (data will be removed if it meets the following requirement: |observation - mean| > W_n_^*^(standard deviation), where W_n_ is the Chauvenet coefficient. Cumulative normal distribution probability of Chauvenet coefficient is 1–1/(4*n), where n is sample size (Ni et al., 2019)), or abnormal data revealed by diagnostic plot of PPK model, and the absolute value of weighted residual (|WRES|) > 5.

A total of 39 covariates were collected, including demographic data, vital signs, hematology tests, serum chemistry, disease status, food, concomitant medications, and clinical study center. The serum creatinine was measured using enzymatic method, and creatinine clearance (CL_cr_) was calculated by Cockcroft Gault formula. The missing data of covariates were imputed by the mean value of other subjects at the same time point (Supplementary Table S2). Some hematology and serum chemistry covariates were converted as follows in order to eliminate the effects of different normal ranges on calculation: measured value/[0.5×(upper limit of normal range + lower limit of normal range)]. The distribution of data was assessed visually based on histogram.

PPK analysis was performed using NONMEM (Version 7.4, ICON PLC, Ireland) and PDxPop software (Version 4.0, ICON PLC, Ireland). Intel Visual Fortran Compiler (Version 11.0, Intel Company, United States). R (Version 2.4.1, R Development Core Team), Xpose (Version 4.0, Uppsala University) and Phoenix WinNonlin (Version 6.0, Certara Company) were used to analyze the export results of PPK calculation. First-order (FO) and first-order conditional estimation with interaction (FOCEI) was used in parameter estimation. Model optimization was determined by objective function value (OBJ) and goodness-of-fit plot.

#### Base model

Compartment modeling based on mean concentration data from phase I trial showed that the PK profile of nemonoxacin in healthy subjects was consistent with two-compartment model, where the absorption was a first-order process. Therefore, it was used as the structure of base model in PPK analysis. Distribution of PK parameters was assumed to conform to lognormal distribution, and the exponential model was used to describe inter-individual variation:

$${Para}_{ind}={Para}_{pop}\times \exp\left(\eta +\kappa \right)$$ (1)

where ${Para}_{ind}$ and ${Para}_{pop}$ were individual and typical values of PK parameter, respectively. *η* denotes inter-individual random effect consistent with normal distribution with mean 0 and variance ω^2^. Since PK parameters of nemonoxacin may change at steady state (72 h following multiple doses) compared to the single dose administration, parameter κ was introduced to describe the variation of PK parameters over time (IOV) (Karlsson & Sheiner, 1993). κ was consistent with normal distribution with mean 0 and variance π^2^_._

Intra-individual variation of PK parameters was consistent with mixed model:

$$Y=Y_{pred}\times \left(1+\varepsilon_{1}\right)+\varepsilon_{2}$$ (2)

where Y and $Y_{pred}$ were measured values and model prediction, respectively. ε indicates intra-individual random effect consistent with normal distribution with mean 0 and variance σ^2^.

#### Fixed effect model

For continuous variables, power (Eq. 3) or linear model (Eq. 4) were used to describe the effects of covariates on PK parameters:

$${Para}_{ind}={Para}_{pop}\times {\left(Cov/Cov_{mean}\right)}^{\theta }$$ (3)

$${Para}_{ind}={Para}_{pop}+\left(Cov-Cov_{mean}\right)\times \theta$$ (4)

Where Cov means covariate, θ is parameter. For categorical variables, the power (Eq. 5) or proportional model (Eq. 6) were used:

$${Para}_{ind}={Para}_{pop}\times \left({Cov}^{\theta }\right)$$ (5)

$${Para}_{ind}={Para}_{pop}\times \left(1+\theta \times Cov\right)$$ (6)

Value of Cov was 0 or 1. During the screening, the covariate model was tested on the PK parameter if one of the followings are met: 1) adding covariate on PK parameter could significantly reduce OBJ in the pre-screening (*p* < 0.05); 2) the relationship between covariate and PK parameter could be explained reasonably; 3) there are some correlation between ETA and covariate in the ETA-covariate plot (*R* ^2^ ≥ 0.02).

Screening of covariates was performed by forward inclusion and backward elimination method. A covariate was included in the model when OBJ reduction was greater than 6.63 (*p* < 0.01) during forward inclusion. Power model, linear model, or proportional models were tested. A covariate was removed when OBJ increase was less than 10.8 (*p* > 0.001) during backward elimination.

Before covariate screening, the following priori information was added in the base model: 1) CL_cr_ was added on CL since nemonoxacin was mainly eliminated by kidney (Guo et al., 2012a); 2) body weight (BW) was added on CL, V2, V3 and Q using power model, because pre-screening of covariates showed that introduction of BW on clearance and distribution volume could significantly reduce OBJ. The power exponent was set as 0.75, 1, 1 and 0.75, respectively, according to the literature (Germovsek et al., 2017; Holford & Anderson, 2017; Sinha et al., 2019). The power base was 70 kg (Germovsek et al., 2019).

#### Model evaluation

Bootstrap analysis was used to evaluate the PPK model. The principle of bootstrap method has been described in our previous paper (Chen Y C et al., 2009). Bootstrap analysis was repeated 300 times. Visual predictive check (VPC) was used to verify PPK model (Wang & Zhang, 2012) using PsN software (Version 5.0.0, Uppsala University), where each subject was simulated 1000 times.

#### Effect of covariates on PK

The effect of renal dysfunction on PK parameters was evaluated because CAP patients with moderate and severe renal dysfunction were not recruited in clinical trials. CL_cr_ was specified as 0, 30, 50, 90, 150 or 200 ml/min, while measured values were used for other covariates. NONMEM was used to simulate the time profile of nemonoxacin following administration of 500 mg q24 h for 10 days. Each subject was simulated 50 times. Based on individual predicted values (IPRE), non-compartment PK parameters at steady state, such as AUC_0-24,ss_, C_max,ss_, T_max_, T_1/2_, CL_ss_/F, V_ss_/F, MRT_0-inf_ and C_min,ss_ were calculated (Wu et al., 2015) using Matlab software (Version 7.0.1, Mathworks Co. Ltd., United States). Changes of PK parameters in patients with moderate and severe renal impairment were evaluated using subjects with normal renal function as control.

For other covariates, similar method was used to obtain the non-compartment PK parameters when the covariate took the specified value. Changes of PK parameters were evaluated to investigate the impact of covariate.

In order to verify that the covariates are indeed clinically significant, randomization test was used. In detail, the values in the randomized covariate column will be shuffled 1000 times, and then the model was run with each of the new dataset. Comparison between randomization test results and results based on original data were performed, including OBJ, estimation time and parameter values. This was implemented using PsN software.

### PK/PD analysis

#### PPK simulation

The PK profile of nemonoxacin in all the subjects participating in phase II-III clinical trials was simulated using their actual dosing regimen in the trial. Each subject was simulated 100 times. AUC_0-24_ and C_max_ of nemonoxacin at steady state were calculated.

#### Exposure-response analysis

The pathogenic bacteria were isolated from the blood or sputum cultures of CAP patients in phase II/III clinical trials. The minimum inhibitory concentration (MIC) of nemonoxacin was determined using the microdilution method recommended by the Clinical and Laboratory Standards Institute. MIC data were analyzed in terms of MIC_50_, MIC_90_, and distribution frequency.

The CAP patients with baseline isolate and efficacy data were selected to develop the PK/PD dataset. The PK/PD indices including AUC_0-24_/MIC, C_max_/MIC, and %T > MIC were calculated. The correlation between these PK/PD indices and efficacy was analyzed. Logistic regression, classification and regression tree (CART), receiver operating characteristic (ROC) curve, and contingency table were used to perform exposure-response analysis. The model for logistic regression was shown as follows [using Ln (AUC_0-24_/MIC)∼Clinical effect as the example]:

$$\left\{\begin{array}{l} Logit=Slope\cdot \mathrm{Ln}\left(AUC_{0-24}/MIC\right)+Intercept\\ P_{Clinical\;effect}\left(\%\right)=\frac{1}{1+e^{-Logit}}\times 100\% \end{array}\right.$$ (7)

If the *p*-value for slope [the test statistic is Wald χ^2^, the formula is χ^2^=(slope/standard error (slope))^2^] is lower, the significance of the logistic regression model is higher.

PK/PD target was searched as follows: the range of PK/PD index was divided by 1000; after contingency table analysis, the area with *p* ≤ 0.05 was found. Within these areas, PK/PD target was further searched as follows: 1) *p*-value obtained from logistic regression was ≤0.05; 2) Youden index from ROC analysis [It both measures the effectiveness of a diagnostic marker and enables the selection of an optimal threshold value (cutoff point). Youden index = sensivity + specificity-1 (Fluss et al., 2005)] was the highest; 3) potential PK/PD target could be found by CART analysis. Lastly, PK/PD index (continuous variable) was converted to binary variable according to potential target, and subjected to logistic regression. The PK/PD target was confirmed when *p*-value was still ≤0.05.

In addition, putting the PK/PD target of nemonoxacin against various bacteria together, the correlation between PK/PD target and MIC data was analyzed using linear model. The MIC data means MIC_50_, or MIC_90_ or base-2 logarithm of them.

#### Monte Carlo simulation (MCS)

Probability of target attainment (PTA) and cumulative fraction of response (CFR: the probability for PK/PD index reach the target against specific bacteria with various MIC level) for two dosing regimens (500 mg or 750 mg, q24 h, 10 days) of nemonoxacin were evaluated by MCS using Matlab software as previously reported (Cao et al., 2013), in which the PK/PD target was obtained from exposure-response analysis.

## Results

### Fundamental data

Overall, 161 subjects (195 cases) were enrolled in phase I to III clinical trials of nemonoxacin, including 125 CAP patients. A total of 2027 plasma concentrations were available from these subjects for building the PPK dataset of nemonoxacin (Supplementary Table S1). The baseline characteristics of subjects were summarized in Table 1. Significant difference was found for some parameters between healthy subjects and CAP patients (Supplementary Table S3): 1) heart rate and body temperature increased; 2) white blood cell count and neutrophils increased, but the hemoglobin reduced by 6.4%; 3) alanine aminotransferase, *γ*-glutamyl transpeptidase, creatine kinase, serum creatinine and glucose increased, while the albumin decreased by 14% (all *p* < 0.01).

**TABLE 1:** Baseline characteristics of subjects in phase I to III clinical trials of nemonoxacin.

	CAP patients (*n* = 125)	Healthy subjects (*n* = 36)	Total (*N* = 195)
Sex (male/female)	77/48	18/18	111/84
Age (years)	39.5 ± 15.3 (36.6, 18-70)	24.3 ± 3.9^*^ (23.5, 18-35)	34.0 ± 14.4 (27.5, 18-70)
Height (cm)	166.0 ± 8.5 (165.9, 150-187)	166.8 ± 6.7 (165.0, 154-179)	166.3 ± 7.7 (165, 150-187)
Body weight (kg)	61.9 ± 9.8 (61.0, 42-90)	59.6 ± 7.2 (57.5, 50-78)	61.0 ± 9.0 (60, 42-90)
Body mass index (kg/m^2^)	22.5 ± 3.2 (22.2, 18.0-33.3)	21.4 ± 1.5^*^ (21.0, 19.1-24.6)	22.1 ± 2.8 (21.7, 18.0-33.3)
CL_cr_ (mL/min)^a^	103.8 ± 27.9 (100.4, 50.7-187.5)	130.0 ± 17.3^*^ (127.2, 100.9-185.3)	114.4 ± 29.2 (116.2, 50.7-200.7)
Albumin ^d^	0.97 ± 0.09 (0.98, 0.76-1.24)	1.13 ± 0.05^*^ (1.13, 0.99-1.22)	1.04 ± 0.12 (1.03, 0.76-1.24)
Hemoglobin ^d^	0.98 ± 0.10 (0.99, 0.61-1.30)	1.05 ± 0.09^*^ (1.05, 0.92-1.29)	1.00 ± 0.10 (1.00, 0.61-1.30)
Underlying disease	38 (30.4)	0	NA
Hypertension	6 (12.2)^b^	0	NA
COPD	3 (6.1)^b^	0	NA
Chronic bronchitis	3 (6.1)^b^	0	NA
Emphysema	3 (6.1)^b^	0	NA
Disease history	12 (9.6)	0	NA
Concomitant medication	55 (44.0)	0	NA
Ambroxol	36 (38.3)^c^	0	NA
Licorice	5 (5.3)^c^	0	NA
Codeine oral liquid	4 (4.3)^c^	0	NA
Food (Yes/No)	80/45	11/25**	91/104

Data are presented as mean ± SD (median, range) or number (%) unless otherwise specified.

^a^ CL_cr_ was estimated by Cockcroft-Gault formula

^b^ Percentage = frequency/(total frequency of underlying disease 49 cases)×100%

^c^ Percentage = frequency/(total frequency of generic name 94 cases)×100%

^d^ Result = measured value/[0.5×(upper limit of normal range + lower limit of normal range)]

*P < 0.01 (*t*-test), **P < 0.01 (chi-square test) vs CAP patients.

Abbreviations: CAP, community-acquired pneumonia; CL_cr_, creatinine clearance; COPD, chronic obstructive pulmonary disease; NA, not applicable.

Finally, 2007 concentrations were included in the PPK database of nemonoxacin after removing the inappropriate data (20 concentrations, 0.987%), e.g., concentration below LLOQ, plasma concentration outliers based on Chauvenet Criterion, or revealed by diagnostic plot, or concentrations from the subjects with poor compliance.

### PPK model

The base PPK model of nemonoxacin was a two-compartment model, where the absorption was a first-order process with a T_lag_. Model fitting improved significantly when inter-occasion variability (IOV) was introduced into clearance (CL), and the result of calculation became stable. Typical values of PK parameters were shown in Table 2. Meanwhile, the IIV (SD) and IOV (SD) of CL were 3.6% and 13%, and the IIV of V_2_ and V_3_ was 5.7% and 8.8%, respectively. The IIV of K_a_ and T_lag_ were 81% and 83%. IIV of bioavailability (F) was 19%. The proportional and additive errors were 0.18 and 0.0062, respectively.

**TABLE 2:** Parameter estimates for final population pharmacokinetic model of nemonoxacin.

Parameter	Unit	Description	Estimate based on original dataset	Estimate based on bootstrap dataset^a^		Bias (%)
			Mean (shrinkage%)	Mean	RSE (%)	
CL	L/h	Clearance	10.3	10.4	1	0
V2	L	Central volume of distribution	103	103	1	0
Q	L/h	Inter-compartment clearance	2.0	2.0	2	1
V3	L	Peripheral volume of distribution	28	28	1	0
KA	1/h	Absorption rate	2.2	2.3	11	6
TLAG	h	Lag time in absorption	0.19	0.18	8	-2
CL_cr_ (CL)	1000/60	Impact factor of CL_cr_ on CL	0.026	0.026	3	1
Food (TLAG)	NA	Power exponent of food on T_lag_	1.6	1.6	9	2
SEX (V2)	NA	Power exponent of sex on V2	0.89	0.88	1	0
Food (Ka)	NA	Power exponent of food on K_a_	0.44	0.42	8	-4
DisStat (V3)	NA	Power exponent of disease status on V_3_	1.2	1.3	5	2
Food(F)	NA	Power exponent of food on F	0.88	0.88	2	0
$\omega_{CL}^{2}$	NA	Inter-individual variability of CL	0.0013 (75)	0.0014	48	11
$\omega_{V2}^{2}$	NA	Inter-individual variability of V2	0.0034 (71)	0.0031	15	-7
$\omega_{V3}^{2}$	NA	Inter-individual variability of V3	0.0080 (64)	0.0094	63	19
$\omega_{KA}^{2}$	NA	Inter-individual variability of KA	0.64 (29)	0.67	10	4
$\pi_{CL}^{2}$	NA	Inter-occasion variability of CL	0.017 (45 or 20)^b^	0.016	9	-4
$\omega_{TLAG}^{2}$	NA	Inter-individual variability of TLAG	0.71 (39)	0.69	12	-3
$\omega_{F}^{2}$	NA	Inter-individual variability of F	0.035 (27)	0.033	10	-4
$\sigma_{prop}^{2}$	NA	Proportional error	0.032 (16)	0.032	10	-2
$\sigma_{add}^{2}$	10^–4^	Additive error	0.34 (14)	0.33	10	-2

Bias (%) = (Para__bootstrap_dataset_/Para__original_dataset_-1)×100%, Para means parameter estimate.

Parameter values are for a 70 kg adult.

Abbreviations: CL_cr_, creatinine clearance; DisStat, disease status; NA, not applicable; RSE%, relative standard error (%); TLAG, lag time.

^a^ 23 runs were successful in the bootstrap analysis.

^b^ 45 is for occasion 1 (time≤72 h), 20 is for occasion 2 (time>72 h).

Age, bilirubin and concomitant medication were not included in the final model due to their variable effects on PK parameters. Using FO algorithm, seven covariates (CL_cr_, weight, sex, albumin, hemoglobin, food, and age) were preliminarily identified as significant covariates of PK parameters. Further screening by FOCEI algorithm confirmed that five covariates (CL_cr_, body weight, DisStat, sex, and food) were significant covariates of nemonoxacin PK parameters: CL_cr_ was the covariates on CL, while food was the covariate on K_a_, T_lag_ and F. Sex was the covariate on V2. Disease status (DisStat) was the covariate on V_3_. Body weight was the covariate on CL, V_2_, Q and V_3_.

The final PPK model was as follows, and parameter estimates are presented in Table 2.

$$\begin{array}{l} CL=\left(10.3+0.026\times CL_{cr}\right)\times {\left(\frac{BW}{70}\right)}^{0.75}\times e^{\eta_{1}+\kappa }\left(L/h\right)\\ V2=103\times \frac{BW}{70}\times 0.89^{Sex}\times e^{\eta_{2}}\left(L\right)\\ Q=2.0\times {\left(\frac{BW}{70}\right)}^{0.75}\left(L/h\right)\\ V3=28\times \frac{BW}{70}\times 1.23^{DisStat}\times e^{\eta_{3}}\left(L\right)\\ KA=2.2\times 0.44^{Food}\times e^{\eta_{4}}\left(1/h\right)\\ TLAG=0.19\times 1.6^{Food}\times e^{\eta_{5}}\left(h\right)\\ F1=1\times 0.88^{Food}\times e^{\eta_{6}} \end{array}$$ (8)

Where CL_cr_ denotes creatinine clearance. Value of sex was 0 for male and 1 for female. Value of food was 1 if taking food within 2 h before administration or within 30 min after administration, otherwise the value was 0. DisStat indicates disease status, 0 for healthy subjects and 1 for CAP patients.

In the backward elimination, the OBJ increased by 45, 33 and 16 if KA-Food, TLAG-Food or F1-Food was removed from final model, respectively. Meanwhile, the OBJ increased by 25 and 46 respectively, if V3-DisStat or V2-Sex was removed from the model. This suggested that these relations have significant impact on reducing OBJ of PPK model.

Final PPK model of nemonoxacin demonstrated that 1) clearance of nemonoxacin increased with CL_cr_; 2) central volume of distribution (V_2_) decreased by 11% in female subjects compared to male subjects; 3) absorption rate of nemonoxacin under fed condition was only 44% of that under fasting condition. Meanwhile, the lag in absorption time increased by 60%, and the absolute bioavailability reduced by 12%; 4) peripheral volume of distribution (V_3_) increased by 23% in CAP patients compared to healthy subjects; 5) CL, V_2_, Q and V_3_ increased along with body weight.

**FIGURE 1:**
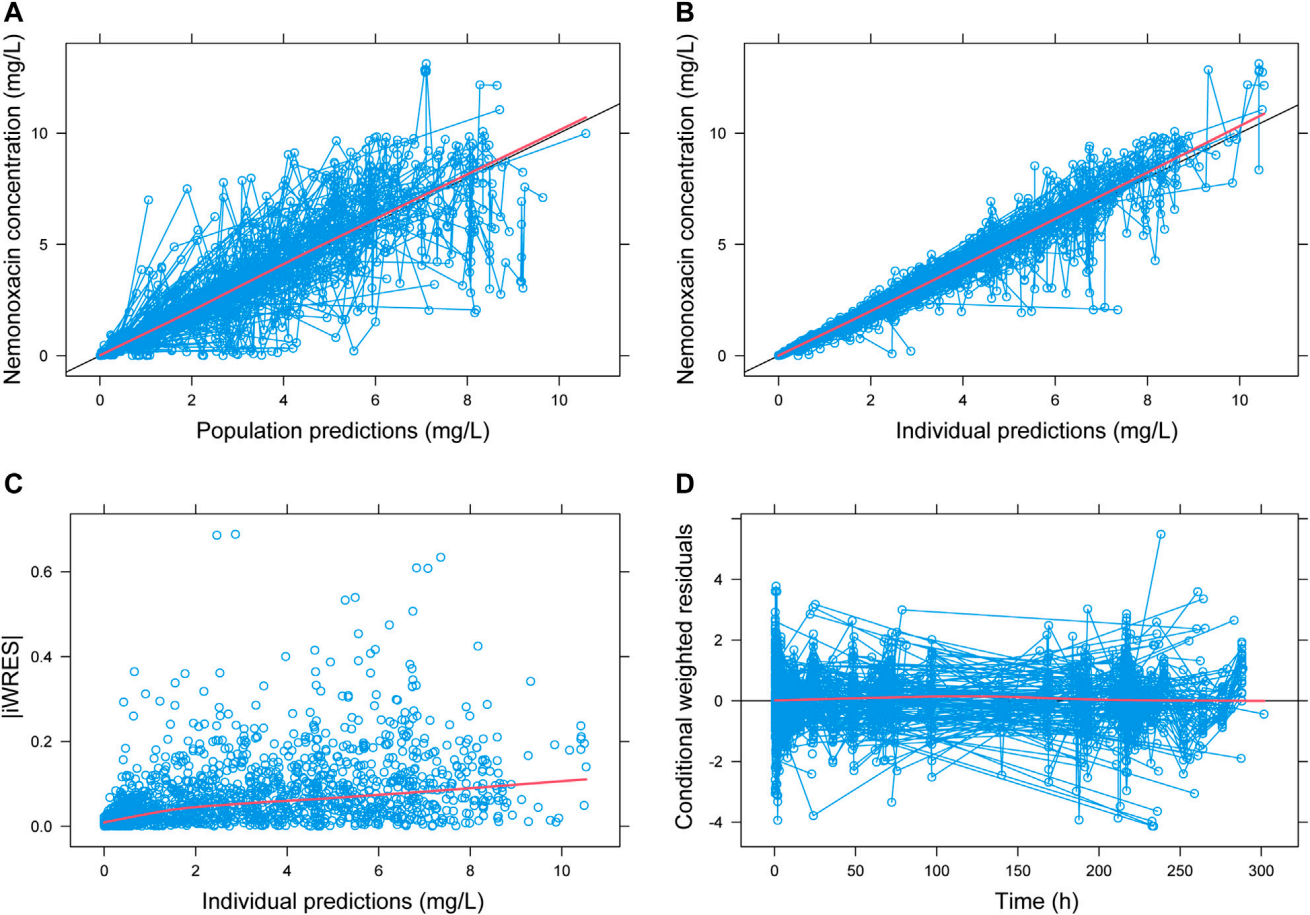
Goodness-of-fit plot for population PK model of nemonoxacin. iWRES: individual weighted residual. Blue circle: actual data. Black line: unity line **(A,B)** or zero horizontal line **(D)**. Red line: locally weighted linear regression.

Based on the final PPK model, the correlation between population predicted values (PRED), IPRE, and observed concentrations (dependent variable, DV) are illustrated in Figure 1. Correlation coefficient (*R* ^2^) was 0.79 and 0.95, indicating that the model explained most of the variances among PK data. Correlation between conditional weighted residuals (CWRES) and TIME was also shown in Figure 1. The data distributed evenly across the zero-horizontal line, and the correlation coefficient was very low (*R* ^2^ = 2E-5). Histogram showed that the CWRES was consistent with normal distribution, while the PK parameters and their IIVs were consistent with log-normal and normal distribution, respectively. Correlations among the PK parameters of nemonoxacin were shown in Supplementary Table S4: 1) 4 pairs had high correlation (*R* ^2^ > 0.7): V2-Q (*R* ^2^ = 0.91), F-F_IIV (*R* ^2^ = 0.83), T_lag_-T_lag__IIV (*R* ^2^ = 0.79) and V3-Q (*R* ^2^ = 0.73). 2) 8 pairs had medium correlation (0.3 < *R* ^2^ ≤ 0.7). For example, V2-V3 (*R* ^2^ = 0.68), CL-Q (*R* ^2^ = 0.60), KA-KA_IIV (*R* ^2^ = 0.58) and CL_IIV-CL_IOV2 (*R* ^2^ = 0.63). 3) Other pairs had low correlation (*R* ^2^ ≤ 0.3).

Bayesian analysis based on PPK model showed that nemonoxacin AUC_0-24_ was 42.0 and 65.5 mg·h/L, respectively after administration of 500 mg or 750 mg, which was close to the actual AUC_0-24_ (46.9 mg·h/L at dose of 500 mg, 62.5 mg·h/L at dose of 750 mg) in CAP patients. The predicted C_max_ was 5.00 and 7.50 mg/L following dose of 500 or 750 mg, which was also close to the actual C_max_ (5.42 mg/L at dose of 500 mg, 8.49 mg/L at dose of 750 mg) in CAP patients. These findings further confirmed the robustness of PPK model in predicting the exposure of nemonoxacin in CAP patients. Bayesian analysis predicted the AUC_0-24_ and C_max_ in healthy subjects (37.8 mg·h/L and 4.94 mg/L) slightly lower than the actual AUC_0-24_ (42.4 mg·h/L) and C_max_ (5.91 mg/L). This may be due to the inclusion of relevant covariates (such as sex) in the PPK model.

PK parameter estimates based on bootstrap dataset were close to that based on original dataset (Table 2). The range for relative bias was (-7%, 19%), most of the relative bias were within the range (-4%, 4%), indicating that the PPK model estimates were reliable. Based on bootstrap dataset, histograms demonstrated that: 1) 3 PK parameters (V3, F1-Food and V2-Sex) were consistent with normal distribution; 2) 12 PK parameters (such as CL, V2 and Ka-IIV) were consistent with left skewness; 3) 6 PK parameters (such as V2-IIV, CL-IOV and σ_add_) were consistent with right skewness (Supplementary Figure S1).

VPC results for the final PPK model are shown in Supplementary Figure S2. Most of the concentrations after single- and multiple-dose in healthy subjects located in 95% confidence interval (CI) of the simulated values based on PPK model. For CAP patients, the change pattern of plasma concentration was close to that of PPK model simulation. Most of the values located in 95% CI. VPC results for 750 mg and 250 mg of nemonoxacin were similar to that of 500 mg dose.

Effect of renal function on nemonoxacin PK parameters is shown in Supplementary Figures S3–S10. Compared to the subjects with normal renal function (CL_cr_ = 200 ml/min), V_ss_/F and CL_ss_/F of nemonoxacin reduced by 8% and 22%, respectively in subjects with mild renal insufficiency (60 < CL_cr_ ≤ 89 ml/min). C_max,ss_ and AUC_0-24,ss_ increased by 8% and 28%, while T_1/2_ and MRT_0-inf_ increased by 1.3 h (25%) and 2 h (25%), respectively. For CAP patients with renal failure (CL_cr_ ≤ 15 ml/min), C_max,ss_ and AUC_0-24,ss_ of nemonoxacin increased by 13% and 47% respectively compared to the subjects with normal renal function.

As shown in the Supplementary Figures S3–S10, impact of BW on C_max_, AUC_0-24_, CL_ss_/F and V_ss_/F ranks the first in that of all covariates. Compared to the subject with BW 60 kg, C_max_ and AUC_0-24,ss_ reduced by 24% and 19% in the subject with BW 80 kg, respectively. Meanwhile, CL_ss_/F and V_ss_/F increased by 25% and 29%, respectively.

Impacts of food on PK parameters were mainly shown in T_max_, C_max_ and V_ss_/F. Compared to the fasted status, T_max_ of nemonoxacin increased by 1.2 h (65%) in the subject with fed status (Supplementary Figure S5). Meanwhile, C_max_ reduced by 23% (Supplementary Figure S3), and V_ss_/F increased by 24% (Supplementary Figure S8). Impacts of food on other PK parameters were mild.

Generally, impacts of sex and disease status on PK parameters of nemonoxacin were small (change of PK parameters ≤19%). Compared to healthy subjects, C_min,ss_ of nemonoxacin increased by 19% in CAP patients (Supplementary Figure S10), while T_max_ increased by 11% (Supplementary Figure S5). Compared to male subjects, C_min,ss_ reduced by 13% in female subjects.

Compared to final model, the reduction of OBJ decreased obviously after using randomization test (Supplementary Figure S11). OBJ reduction in final dataset was statistically significant lower than that in randomization dataset (*p* < 0.05) (Supplementary Table S5).

### PK/PD analysis

**FIGURE 2:**
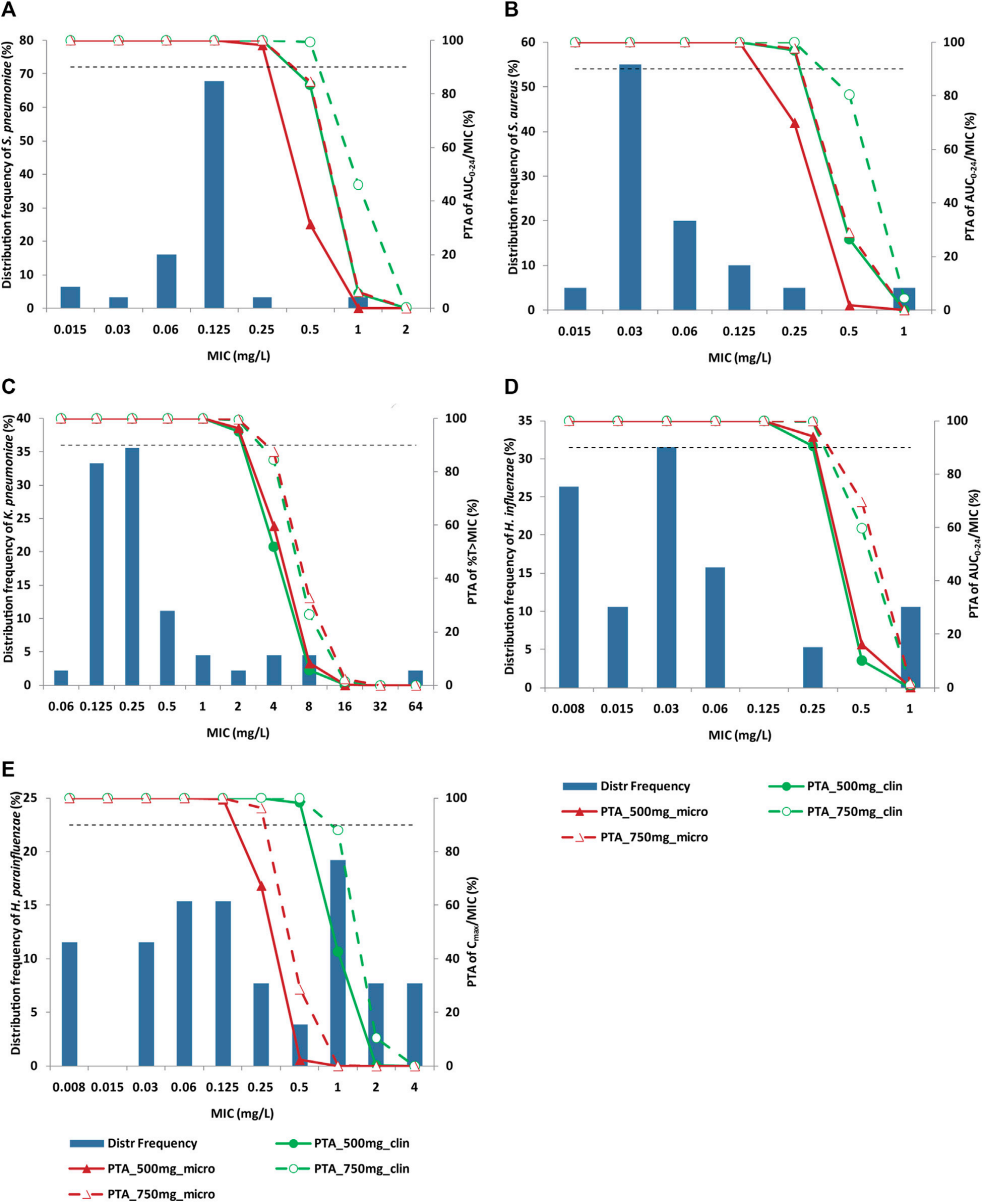
Distribution of nemonoxacin MIC against specific pathogen and PTA of PK/PD index. Panel **(A)** *S. pneumoniae*; Panel **(B)** *S. aureus*; Panel **(C)** *K. pneumoniae*; Panel **(D)** *H. influenzae*; Panel **(E)** *H. parainfluenzae*. Histograms and broken lines correspond to left and right *y*-axis, respectively. Solid line and dash line represent PTA for nemonoxacin 500 mg q24 h and 750 mg q24 h, respectively. Green and dark red colors represent clinical and microbiological targets, respectively. Horizontal dash line denotes 90% PTA. Nemonoxacin was administered q24 h for 10 days. MIC: minimum inhibitory concentration; PK/PD: pharmacokinetic/pharmacodynamic; PTA: probability of target attainment.

Baseline isolate was available for 246 CAP patients. Among them, 158 CAP patients had the efficacy data. A total of 175 strains were included in the evaluation dataset, including *K. pneumoniae* (25.7%), *Haemophilus* (25.7%), *S. pneumoniae* (17.7%) and *S. aureus* (11.4%). MIC distribution of nemonoxacin against CAP pathogens is illustrated in Figure 2. The MIC_90_ of nemonoxacin was both 0.125 mg/L against *S. pneumoniae* and *S. aureus* (MIC range 0.015–1 mg/L for both species). Most of the MIC values (80%) against *K. pneumoniae* were in the range of 0.125–0.5 mg/L (MIC_90_, 4 mg/L). Most of the MIC values (38.5% and 84.2%) for *H. parainfluenzae* and *H. influenzae* were in the range of 0.008–0.06 mg/L (MIC_90_, 2 and 1 mg/L).

**FIGURE 3:**
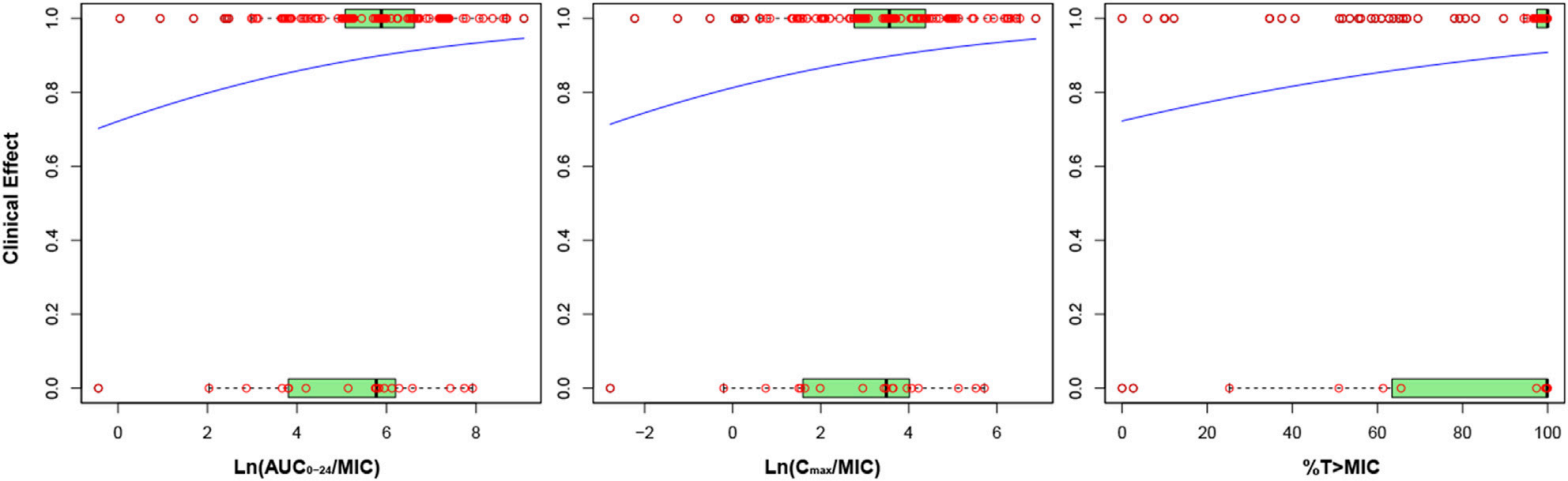
Correlation between nemonoxacin PK/PD index and clinical efficacy. *Y* axis indicates probability of successful clinical efficacy. Red circles indicate actual data, and blue line indicate fitting from the logistic regression model. The box plot with light green characterizes the distribution pattern of actual data: the left limit, inner line and right limit of box are 25%, 50% and 75% percentile of actual data, respectively.

Figure 3 depicts the correlation between nemonoxacin PK/PD index and clinical efficacy. Probability for clinical success increased with the value of PK/PD index. The natural logarithm of AUC_0-24_/MIC and C_max_/MIC correlated with clinical efficacy much better than AUC_0-24_/MIC or C_max_/MIC. *p*-value of slope for Ln (AUC_0-24_/MIC), Ln (C_max_/MIC) and %T > MIC was 0.125, 0.151, and 0.112, respectively.

The correlation between PK/PD index and clinical efficacy was also analyzed in terms of specific pathogen of CAP (Supplementary Table S6, Supplementary Figure S12). Slope for *S. pneumoniae* dataset was negative, and close to zero for *S. aureus* dataset. For *K. pneumoniae*, slope was positive for %T > MIC and the *p*-value was the lowest, suggesting it was time-dependent. For *H. influenzae* and *H. parainfluenzae*, slope for Ln (AUC_0-24_/MIC) and Ln (C_max_/MIC) was positive and the *p*-value was lower than that for %T > MIC, indicating the PK/PD was concentration-dependent.

Nemonoxacin PK/PD targets are summarized in Table 3. Overall, AUC_0-24_/MIC, C_max_/MIC and %T > MIC targets for clinical efficacy were 45.6, 7.32, and 51.01%, respectively. Probability for clinical success was 91% when PK/PD index ≥ corresponding target. AUC_0-24_/MIC, C_max_/MIC and %T > MIC targets were the same for either microbiological efficacy or clinical efficacy. For *S. pneumoniae* and *S. aureus*, the AUC_0-24_/MIC target for clinical efficacy was 63.3 and 97.8, respectively. For *K. pneumoniae*, the %T > MIC target for clinical and microbiological efficacy was 7.96% and 4.31%, respectively. For *H. influenzae*, the AUC_0-24_/MIC target for clinical and microbiological efficacy was 115.7 and 107.8, respectively. C_max_/MIC target for clinical and microbiological efficacy was 4.9 and 16.3 against *H. parainfluenzae*, respectively.

**TABLE 3:** Nemonoxacin PK/PD target against Gram positive and negative bacteria.

Pathogen	AUC_0-24_/MIC target		C_max_/MIC target		%T > MIC target	
	Clinical efficacy	Microbiological efficacy	Clinical efficacy	Microbiological efficacy	Clinical efficacy	Microbiological efficacy
Total	**45.6 (91%)**	**45.6 (89%)**	7.3 (91%)	7.4 (89%)	51.0% (91%)	51.0% (88%)
*S. pneumoniae*	**63.3 (87%)**	**93.7 (86%)**	43.7 (95%)	25.9 (85%)	55.9%	74.7%
*S. aureus*	**97.8**	**143.3**	43.7	25.9	73.3%	100%
*K. pneumoniae*	NA	15.5 (90%)	NA	1.1 (90%)	**8.0% (90%)**	**4.3% (88%)**
*H. influenzae*	**115.7 (88%)**	**107.8 (88%)**	11.1 (88%)	11.1 (88%)	NA	NA
*H. parainfluenzae*	51.4 (94%)	244.7 (93%)	**4.9 (95%)**	**16.3 (94%)**	66.2% (94%)	99.3% (93%)

Percentage in parentheses denotes probability of clinical success or assumed eradication of pathogen when PK/PD index ≥ corresponding target.

Abbreviations: MIC, minimum inhibitory concentration; NA, not available; PK/PD, pharmacokinetic/pharmacodynamic.

Bold numbers are primary PK/PD targets.

The correlations between nemonoxacin PK/PD targets and MIC are depicted in Supplementary Figure S13. When putting the PK/PD target against various bacteria together, PK/PD target reduced as MIC increased. For clinical efficacy, Log_2_ (MIC_50_) showed the best correlation with AUC_0-24_/MIC target (*R* ^2^ = 0.67). For microbiological efficacy, MIC_90_ had the best correlation with C_max_/MIC target (*R* ^2^ = 0.47).

PTA of nemonoxacin PK/PD indices against main CAP pathogens are shown in Figure 2. PK/PD cutoff values (the maximal MIC with PTA≥ 90%) are summarized in Table 4. For *S. pneumoniae* and *S. aureus*, PTA of AUC_0-24_/MIC and C_max_/MIC for 500 mg dose were greater than 90% based on clinical target when MIC ≤0.25 mg/L. For *K. pneumoniae*, when MIC ≤2 mg/L, PTA of %T > MIC was greater than 90% for clinical efficacy. For *H. influenzae*, PK/PD cutoff value of AUC_0-24_/MIC was 0.25 mg/L both for 500 mg and 750 mg q24 h dosing regimen. For *H. parainfluenzae*, PK/PD cutoff value of C_max_/MIC for clinical and microbiological efficacy was 0.5 and 0.125 mg/L, respectively for 500 mg dose of nemonoxacin, and 0.5 and 0.25 mg/L respectively for 750 mg dose.

**TABLE 4:** PK/PD cutoff values of nemonoxacin (mg/L).

Pathogen	PK/PD index	MIC_50_/MIC_90_	Nemonoxacin 500 mg q24 h		Nemonoxacin 750 mg q24 h	
			Clinical efficacy	Microbiological efficacy	Clinical efficacy	Microbiological efficacy
*S. pneumoniae*	AUC_0-24_/MIC	0.125/0.125	0.25	0.25	0.5	0.25
*S. aureus*	AUC_0-24_/MIC	0.03/0.125	0.25	0.125	0.25	0.25
*K. pneumoniae*	%T > MIC	0.25/4	2	2	2	2
*H. influenzae*	AUC_0-24_/MIC	0.03/1	0.25	0.25	0.25	0.25
*H. parainfluenzae*	C_max_/MIC	0.125/2	0.5	0.125	0.5	0.25

PK/PD cutoff was the maximal MIC with probability of target attainment ≥90%.

Abbreviations: MIC, minimum inhibitory concentration; PK/PD, pharmacokinetic/pharmacodynamic.

CFR of nemonoxacin PK/PD indices are presented in Table 5. CFR was >93% against *S. pneumoniae* and *S. aureus* and >91% against *K. pneumoniae* at dose of 500 mg. For *H. influenzae*, CFR of AUC_0-24_/MIC was about 89%–90%. For *H. parainfluenzae*, CFR of C_max_/MIC was 74% for clinical efficacy at 500 mg dose of nemonoxacin, and 83% in case of 750 mg dose.

**TABLE 5:** Cumulative fraction of response of nemonoxacin PK/PD indices.

Pathogen	Nemonoxacin 500 mg q24 h		Nemonoxacin 750 mg q24 h	
	Clinical efficacy	Microbiological efficacy	Clinical efficacy	Microbiological efficacy
*S. pneumoniae*	97	97	98	97
*S. aureus*	95	93	95	95
*K. pneumoniae*	91	92	94	94
*H. influenzae*	89	89	90	90
*H. parainfluenzae*	74	59	83	62

All data are presented as percentage.

PK data were derived from Monte Carlo simulation based on PPK model at steady state in CAP patients receiving nemonoxacin q24 h for 10 days.

Abbreviations: MIC, minimum inhibitory concentration; PK/PD, pharmacokinetic/pharmacodynamic; PPK, population pharmacokinetics.

## Discussion

The cumulative urinary excretion (Ae_0-24_%) of nemonoxacin was 56.1% and 73.59%, respectively following multiple oral doses of 500 mg and 750 mg in healthy subjects (Guo et al., 2012a). The corresponding Ae_0-24_% was 57.61% and 73.38% in CAP patients (Wu, 2012). These findings suggested that nemonoxacin was mainly eliminated *via* kidney, which was consistent with the results of covariate screening for the PPK model of nemonoxacin.

This PPK analysis revealed the PK profile of nemonoxacin consistent with quinolones in CAP patients. PPK study of sitafloxacin indicated that CL_cr_ was covariate on CL in patients with community-acquired respiratory tract infection (CARTI). Taking sitafloxacin with food could reduce absorption rate (K_a_) by 56.7% (Tanigawara et al., 2013). These data were consistent with the results of PPK analysis for nemonoxacin. Another PPK study showed that CL_cr_, ideal weight, fat, age, and pseudoephedrine were the covariate on CL, and sex was covariate on V_d_ of garenoxacin in patients with CARTI (Van Wart et al., 2004). This is also close to the results of nemonoxacin. PPK analysis of gemifloxacin (Owens et al., 2005) and levofloxacin (Zhang et al., 2009) reported that the base model was a two-compartment model for both gemifloxacin and levofloxacin. CL_cr_ was a significant covariate on CL, just like in the PPK model of nemonoxacin.

Monte Carlo simulation based on PPK model showed that AUC_0-24_ and C_max_ of nemonoxacin increased by 44% and 12% in case of CL_cr_ ≤ 15 ml/min compared to normal renal function (CL_cr_ = 200 ml/min). This is similar to the results of PPK analysis for other quinolones in patients with CARTI. Taking sitafloxacin for example, the AUC_0-12_ and C_max_ increased by 2.73 times and 1.71 times respectively in case of reduced CL_cr_ (20 ml/min) compared to the control (CL_cr_ = 75 ml/min) (Tanigawara et al., 2013). Similarly, the AUC_0-24_ of levofloxacin increased by 62.4% and 152.5% when CL_cr_ reduced to 50 and 10 ml/min, respectively compared to the corresponding values in subjects with normal renal function (CL_cr_ = 120 ml/min) (Zhang et al., 2009). The corresponding C_max_ increased by 56.7% and 22.3%, respectively. The authors concluded that it was not necessary to adjust the dose of levofloxacin when CL_cr_ ≥ 50 ml/min.

During covariate screening, OBJ reduced significantly when respiratory rate and neutrophils count (neutrophil) were introduced on CL. Using FO method, the OBJ reduced by 19.3 or 11.0 if adding respiratory rate on CL or V_2_, respectively. For neutrophils, the OBJ reduced by 8.1 if adding it on V_3_. Using FOCEI method, the OBJ reduced by 15.3 or 8.3 if adding neutrophils on CL and T_lag_, respectively. However, considering that 1) the relationship between respiratory rate and drug clearance could not be explained; 2) neutrophil was often used as the PD index in clinical studies (Toffoli et al., 2001; van Hasselt et al., 2013), respiratory rate and neutrophil were not included in fixed effect model. OBJ also reduced significantly when glucose was added on V_1_ or K_a_. However, to our knowledge, glucose was not regarded as a covariate on PK parameter. In addition, introduction of QTc interval, sodium or chloride on K_a_ could reduce OBJ significantly. However, the relationship between these indices and the absorption rate was hard to explain, so these indices were not included in the final PPK model.

As for the ethnic differences in PK, we compared the PK of nemonoxacin in healthy subjects in China and United States because there no reports on PK of nemonoxacin in patients in other country besides China. We summarized two tables (Supplementary Tables S7, S8). For single PK, compared with subjects in United States, the AUC_0-inf_ and C_max_ of nemonoxacin increased by 31% and 73% in Chinese subjects, while the V_d_/F decreased by 27% in Chinese subjects (Supplementary Table S7). As for multiple PK, compared with subjects in United States, the AUC_0-24_ and C_max_ of nemonoxacin at steady state in Chinese subjects elevated by 22% and 25%, while T_1/2_ reduced by 32% (Supplementary Table S8). The main reason for the difference of PK parameter maybe the body weight: compared with subjects in United States, the body weight of Chinese subjects decreased by 19% and 25% in single and multiple PK study, respectively. After correction by body weight, the difference of V_d_/F between China and United States population reduced to 11%, while CL/F in China population was equal to that in United States population (Supplementary Table S7). We searched the reports on race difference of PK parameters of quinolones. Results showed that the PK of levofloxacin, moxifloxacin and ciprofloxacin do not show race difference (Fish & Chow, 1997; North et al., 1998; Hasunuma et al., 2016; Kaneko et al., 2018; Tolentino-Hernandez et al., 2020). For each drug, the PK parameters in various races get close to each other after correction by weight (Fish & Chow, 1997; Kaneko et al., 2018; Tolentino-Hernandez et al., 2020). Hence, we predict that the probability for race difference on PK of nemonoxacin in CAP patients is low.

Our PPK model may predict the probability for adjustment of nemonoxacin dosing regimen in the CAP patients with severe renal impairment. As shown in Supplementary Table S9, compared to the patients with normal renal function (CL_cr_ = 250 ml/min), the probability for AUC_0-inf_ ratio (AUC_0-inf_CLcr=0_/AUC_0-inf_control_) greater than 2 was 40% in the CAP patients with CL_cr_ = 0 ml/min. This probability was 13% if selecting the patients with CL_cr_ = 90 ml/min as control. The clinical trial of nemonoxacin in patients with severe renal adjustment showed that, four of ten patients with severe renal impairment [mean CL_cr_: 21 ml/min] had AUC_0-inf_ ratio ≥2 compared to the patients with normal renal function (mean CL_cr_: 106 ml/min) (Li et al., 2020; Li Y et al., 2021). This percentage (40%) was similar to the simulation results from the PPK model.

The *f*AUC_0-24_/MIC and *f*C_max_/MIC targets of nemonoxacin derived from *in vitro* data (Liang et al., 2013), animal studies (Li X et al., 2021) and clinical studies were compared. The *f*AUC_0-24_/MIC target based on *in vitro* data (47.05) was close to the clinical target (0.84 × 63.3 = 53.2, 0.84 denotes free fraction of nemonoxacin). Clinical target of *f*AUC_0-24_/MIC (53.2) was also close to that from animal studies (44.4, 2-log reduction). However, the clinical target of *f*C_max_/MIC (0.84 × 43.7 = 36.7) was higher than that based on *in vitro* data (5.07, 3-log reduction) or animal data (about 20, Figure 2 in (Li X et al., 2021). This may be due to the prediction from the model (Supplementary Figure S13) because all isolates of *S. pneumoniae* were eradicated after multiple doses of nemonoxacin.

Previously, we analyzed the PK/PD data of nemonoxacin using the reported AUC/MIC (Frei & Burgess, 2005; Owens et al., 2005; Louie et al., 2009) and C_max_/MIC targets of quinolones (Kiser et al., 2006; Yoshida et al., 2011) against *S. pneumoniae* in literature. PK/PD cutoff values were dependent on target level: PK/PD cutoff value was 1 and 0.5 mg/L when AUC/MIC target was 25 and 63, respectively. PK/PD cutoff value was 0.5 mg/L when the target from *in vitro* PK/PD study (*f*AUC_0-24_/MIC = 47.05) was used (Liang et al., 2013). Cutoff value was the same for both 500 mg and 750 mg dosing regimens. PK/PD analysis showed similar results for C_max_/MIC and AUC/MIC. Hence, the results of PK/PD analysis based on *in vitro* PK/PD target of nemonoxacin were more reliable than the results based on the target reported in literature.

PK/PD cutoff value of nemonoxacin was 0.25 mg/L against *S. pneumoniae*. MIC_90_ of *S. pneumoniae* was 0.125 mg/L. Hence, *S. pneumoniae* was susceptible to nemonoxacin treatment because PK/PD cutoff value was higher than the MIC_90_. This is similar to the results of PK/PD analysis in phase I trial (Wu et al., 2015): PK/PD cutoff value for *S. pneumoniae* was 0.361 (500 mg) or 0.722 mg/L (750 mg). MIC_90_ of *S. pneumoniae* was 0.043 (penicillin-resistant *S. pneumoniae*) or 0.090 mg/L (penicillin-susceptible and -intermediate *S. pneumoniae*). Thus, nemonoxacin 500 mg is adequate for treatment of CAP in adult patients. PK/PD cutoff value of nemonoxacin was 2 mg/L against *K. pneumoniae*. MIC_90_ of *K. pneumoniae* was 4 mg/L. From the viewpoint of cutoff, 500 mg dose q24 h is not enough for treatment of CAP caused by *K. pneumoniae*, while 750 mg nemonoxacin q24 h is expected to achieve good clinical and microbiological efficacy because PTA (86%) was closed to 90% at MIC = 4 mg/L.

We attempted to analyze the correlation between nemonoxacin PK/PD index and treatment efficacy in treating the CAP caused by *P. aeruginosa*. PK/PD was both time- and concentration-dependent, and %T > MIC and AUC_0-24_/MIC targets were 47% and 30.0, respectively. For clinical efficacy, PK/PD cutoff value was 0.5 mg/L at dose of 500 mg and 1 mg/L at dose of 750 mg. CFR against *P. aeruginosa* was 66.8% at dose of 500 mg and 81.4% at dose of 750 mg. However, *P. aeruginosa* as pathogen was isolated from only 8 CAP patients, more data are required to confirm the clinical significance of this finding for *P. aeruginosa* infection.

In clinics, there are subjects infected by multiple bacteria. However, the percentage of these subjects is relatively low. For nemonoxacin, the percentage of CAP patients infected by multiple bacteria was 11% (17/175) in the phase II and III clinical trial. Most of CAP patients (89%) were infected by single bacteria. Since the aim of this paper was to obtain the PK/PD target against each bacteria, we did not consider the calculation of PK/PD target for the subjects infected by multiple bacteria simultaneously. If one patient was infected by two bacteria simultaneously, we treated him as ‘two patients’, where one patient was infected by one bacteria.

For the safety of nemonoxacin, phase II study showed that, when nemonoxacin dose was 750 mg, the occurrence of treatment-related adverse events (TRAE) was 35.9% (21/59), which mainly includes: nausea (10.2%), leukopenia (10.2%), vomiting (6.8%), abnormal liver function (5.1%) and QT interval prolongation (5.1%) (Liu et al., 2017). The TRAE occurrence for 750 mg dose was similar to that of 500 mg dose in phase II clinical trial (30.6%). Hence we believe that the safety of nemonoxacin is acceptable when the nemonoxacin dose increases from 500mg to 750 mg for the treatment of infection caused by *H. parainfluenzae*.

This integrative PK/PD study added some new knowledge to the understanding of nemonoxacin in CAP patients. Specifically, nemonoxacin PK/PD target was proposed against *S. aureus* and *K. pneumoniae*, *H. parainfluenzae*, and *H. influenzae*. Monte Carlo simulation supported that higher nemonoxacin dose (750 mg) was effective for the CAP caused by *H. parainfluenzae*. A new algorithm was proposed and successfully used to derive PK/PD target by combining logistic regression or CART with contingency table, which is more objective because it is not necessary to arbitrarily set the anticipated pharmacological level like using logistic regression alone.

Some limitations may affect the interpretation of our findings. First, the PK/PD target for *S. pneumoniae* and *S. aureus* was not derived from actual data. All isolates of *S. pneumoniae* and *S. aureus* were eradicated after multiple doses of nemonoxacin. Hence, we predicted the PK/PD target for *S. pneumoniae* and *S. aureus* by linear regression in dataset (PK/PD target vs. MIC_50_ or MIC_90_). Second, patients ≥75 years of age or with moderate/severe renal impairment had been excluded from the clinical trials of nemonoxacin, this PK/PD analysis is not applicable for such patients. Third, we used data from patients with or without mild renal dysfunction and simulate the PK for patients with moderate or severe renal dysfunction (Supplementary Figures S3–S10). This extrapolation is far outside the range of observed CL_cr_. It is necessary to validate the PK predictions of nemonoxacin in CAP patients with moderate or severe renal dysfunction in the future. Fourth, clinical study in patients with severe renal impairment (Li Y et al., 2021) is very important because nemonoxacin PK may change significantly in case of severe renal impairment. When more data of nemonoxacin are available from studies in special populations (Kang et al., 2019; Li Y et al., 2021), we will perform more comprehensive population PK/PD analysis.

## Conclusion

Population PK/PD study of nemonoxacin in Chinese adult patients with CAP confirmed that the PK profile was consistent with a two-compartment model. AUC_0-24_/MIC and %T > MIC were the best PK/PD indices for predicting clinical efficacy of nemonoxacin. The dosing regimen of nemonoxacin 500 mg q24 h for 7–10 days is effective in treatment of CAP caused by *S. pneumoniae*, *S. aureus* and *K. pneumoniae*, irrespective of patient sex, mild renal impairment, empty stomach or not. However, nemonoxacin 750 mg q24 h would provide better efficacy than 500 mg q24 h for the CAP caused by *H. parainfluenzae* in terms of CFR.

## Data Availability

The original contributions presented in the study are included in the article/Supplementary Material, further inquiries can be directed to the corresponding author.

## Supplementary material

Supplementary Figure S1 Histogram of PK parameters of nemonoxacin based on bootstrap dataset.

Supplementary Figure S2 Visual predictive check for the final PPK model of nemonoxacin.

Supplementary Figure S3 Effect of covariate on C_max_ of nemonoxacin at steady state.

Supplementary Figure S4 Effect of covariate on AUC_0-24_ of nemonoxacin at steady state.

Supplementary Figure S5 Effect of covariate on T_max_ of nemonoxacin at steady state.

Supplementary Figure S6 Effect of covariate on T_1/2_ of nemonoxacin at steady state.

Supplementary Figure S7 Effect of covariate on CL/F of nemonoxacin at steady state.

Supplementary Figure S8 Effect of covariate on V/F of nemonoxacin at steady state.

Supplementary Figure S9 Effect of covariate on MRT_0-inf_ of nemonoxacin at steady state.

Supplementary Figure S10 Effect of covariate on C_min_ of nemonoxacin at steady state.

Supplementary Figure S11 Change in OBJ (deltaOBJ) for randomization test of final PPK model of nemonoxacin.

Supplementary Figure S12 Correlation between nemonoxacin PK/PD index and clinical efficacy against each bacteria.

Supplementary Figure S13 Correlations between nemonoxacin PK/PD target and MIC against various pathogens.

Supplementary Table S1 Summary of the phase I to III clinical trials of nemonoxacin capsule.

Supplementary Table S2 Summary of the imputation for the missing covariate data.

Supplementary Table S3 Baseline of the covariates for vital sign, blood routine and biochemistry.

Supplementary Table S4 Correlation of PK parameters for the final PPK model of nemonoxacin (R^2^).

Supplementary Table S5 Summary of deltaOBJ (dOBJ) in the randomization test.

Supplementary Table S6 Correlation between nemonoxacin PK/PD index and clinical efficacy for CAP caused by specific pathogen.

Supplementary Table S7 PK parameter of nemonoxacin in healthy subjects following single oral administration of 500 mg nemonoxacin capsule.

Supplementary Table S8 PK parameter of nemonoxacin at steady state in healthy subjects following multiple oral administration of 500 mg nemonoxacin capsule.

Supplementary Table S9 Probability for ratio of AUC_0-inf_ of nemonoxacin greater than 2 in CAP patients with CL_cr_=0 mL/min.
