# Supplementary material for: Integrative population pharmacokinetic/pharmacodynamic analysis of nemonoxacin capsule in Chinese patients with community-acquired pneumonia
Source: Front Pharmacol. 2023 Feb 28;14:912962. doi: 10.3389/fphar.2023.912962 (PMC10010492; doi:10.3389/fphar.2023.912962)
Supplement: Supplementary file 1 [file DataSheet1.pdf]

**Supplementary Material**

**Title: Integrative Population Pharmacokinetic/Pharmacodynamic Analysis of Nemonoxacin Capsule in Chinese Patients with Community-acquired Pneumonia**

## Inclusion and exclusion criteria for healthy volunteers and CAP patients participating in clinical trials of nemonoxacin

**Healthy volunteers** were enrolled in phase I clinical trial of nemonoxacin if they met all of the following inclusion criteria: (1) 18-45 years of age; (2) BMI, 19-25 kg/$m^2$. Body weight at least 50 kg for males and at least 45 kg for females; (3) No tobacco or nicotine use within 3 months; (4) Willing not to take any foods or beverages containing caffeine or xanthine within 24 hours. The subjects were excluded if they had any of the following exclusion criteria: (1) History of diabetes mellitus, central nervous system disease or other diseases, family history of genetic disease; (2) History of surgery or trauma within 6 months; (3) Receiving Chinese herbal medicine within 14 days or vitamins within 7 days; (4) Received liver enzyme inducer or inhibitor within 30 days, or received any investigational drug within 3 months; (5) Abnormal ECG, or QTc > 450 ms.

**CAP patients** were enrolled in population pharmacokinetic (PPK) study during phase II/III clinical trials if they met all of the following inclusion criteria: (1) 18-70 years of age, body weight 40-100 kg and BMI > 18 kg/$m^2$; (2) Met diagnostic criteria of CAP, i.e., had at least 3 of the following signs/symptoms, one of which must be a or b: a. fever (oral temperature > 37.3℃, rectal temperature > 37.8℃, axillary temperature > 36.8℃); b. white blood cell count (WBC) 1000-4000/$mm^3$, or neutrophils > 70%; c. cough with purulent sputum, dyspnea or shortness of breath; d. chest pain or discomfort; e. signs of lung consolidation (abnormalities on auscultation, such as bronchial sound or local moist rales). (3) Chest X-ray revealed new inflammatory infiltration or infiltration opacity; (4) Suitable samples were available, (including sputum, blood and urine samples) for culture and identification of bacterial strains.

The subjects were excluded if they had any of the following exclusion criteria: (1) Severe CAP, i.e., presence of a or b, or at least 3 of the other signs/symptoms: a. requiring invasive mechanical ventilation; b. septic shock requiring vasoconstrictors; c. respiratory rate ≥ 30 times/min, chest X-ray showed infiltration in multiple pulmonary lobes; d. blood urea nitrogen ≥ 20 mg/dL, WBC < 4000/$mm^3$, platelet ≤ $10^5$/$mm^3$; e. hypothermia (< 36℃), hypotension requiring rehydration, hypoglycemia for non-diabetic patients; f. hyponatremia, or unexplained metabolic acidosis; g. acute alcoholism, cirrhosis or asplenia syndrome. (2) Hospital-acquired infection; (3) Viral pneumonia, aspiration pneumonia, or nosocomial pneumonia (including ventilator associated pneumonia); (4) History of structural lung diseases such as bronchiectasis, cystic fibrosis, bronchial obstruction except chronic obstructive pulmonary disease; (5) History of prolonged QTc interval, or receiving drugs for treatment of prolonged QTc interval; (6) Renal insufficiency or serum

creatinine ⩾ 1.1 times the upper limit of normal (ULN); (7) Alanine or aspartate aminotransferase ⩾ 3 times ULN within 48 h before enrollment; total bilirubin or direct bilirubin ⩾ 1.1 times ULN; (8) Neutrophil count < 1500/$mm^3$ within 48 h before enrollment; (9) Received antibacterial agents for more than 24 hours within 72 hours before enrollment, or use of quinolones within 14 days and had history of allergy to these antibacterial agents; (10) Received other investigational drugs within 30 days before enrollment.

**Efficacy evaluation**

**Clinical efficacy**

**Clinical success**: all symptoms and signs related to pneumonia disappeared. Results of chest X-ray and laboratory tests normalized after treatment and antibacterial therapy was no longer needed. Clinical success was also considered if the persistent clinical symptoms, signs, or laboratory abnormalities after therapy were part of the underlying disease rather than active infection.

**Clinical failure** was considered if a subject satisfied any of the following: (1) main symptoms or signs persisted or did not disappear completely or became worse after therapy; (2) emergence of new symptoms or signs related to pneumonia, or requiring treatment other than nemonoxacin; (3) had to switch to other antimicrobial treatment even though symptoms or signs were improved; (4) received other antimicrobial agents during the period from Visit 3 to Visit 4; (5) discontinued study drug therapy within 3 days after initiation of treatment due to adverse events.

**Indeterminate** was considered if a subject satisfied any of the following: (1) lacking post-treatment evaluation data; (2) additional antibacterial agents were used for other indication, which was not specified by study protocol; (3) discontinued study drug therapy due to reasons unrelated to nemonoxacin.

**Microbiological efficacy**

Microbiological efficacy was evaluated at Visit 3 (end of treatment) and Visit 4 (post-treatment follow-up Visit), which was recorded as: (1) **eradication**: baseline pathogen was negative in sputum culture at Visit 4; (2) **assumed eradication**: the disease under study was cured in the opinion of clinicians, but no sputum was available for culture; (3) **assumed persistence**: no sputum was available for culture in patients who were considered clinical failure; (4) **persistence**: baseline pathogen still positive in sputum culture at post-treatment follow-up Visit; (5) **Indeterminate**: microbiological efficacy could not be evaluated, e.g., follow-up visit was not performed on time.

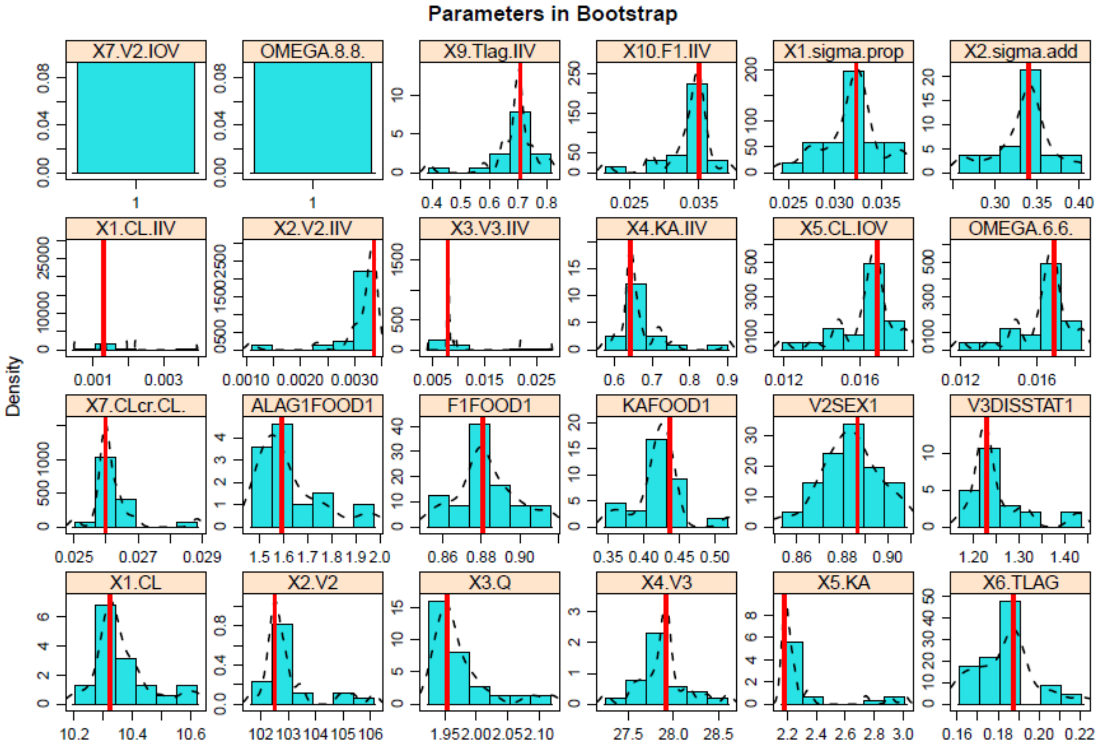


**SUPPLEMENTARY FIGURE 1 |** Histogram of PK parameters of nemonoxacin based on bootstrap dataset. Bootstrap analysis of final PPK model was repeated 200 times.

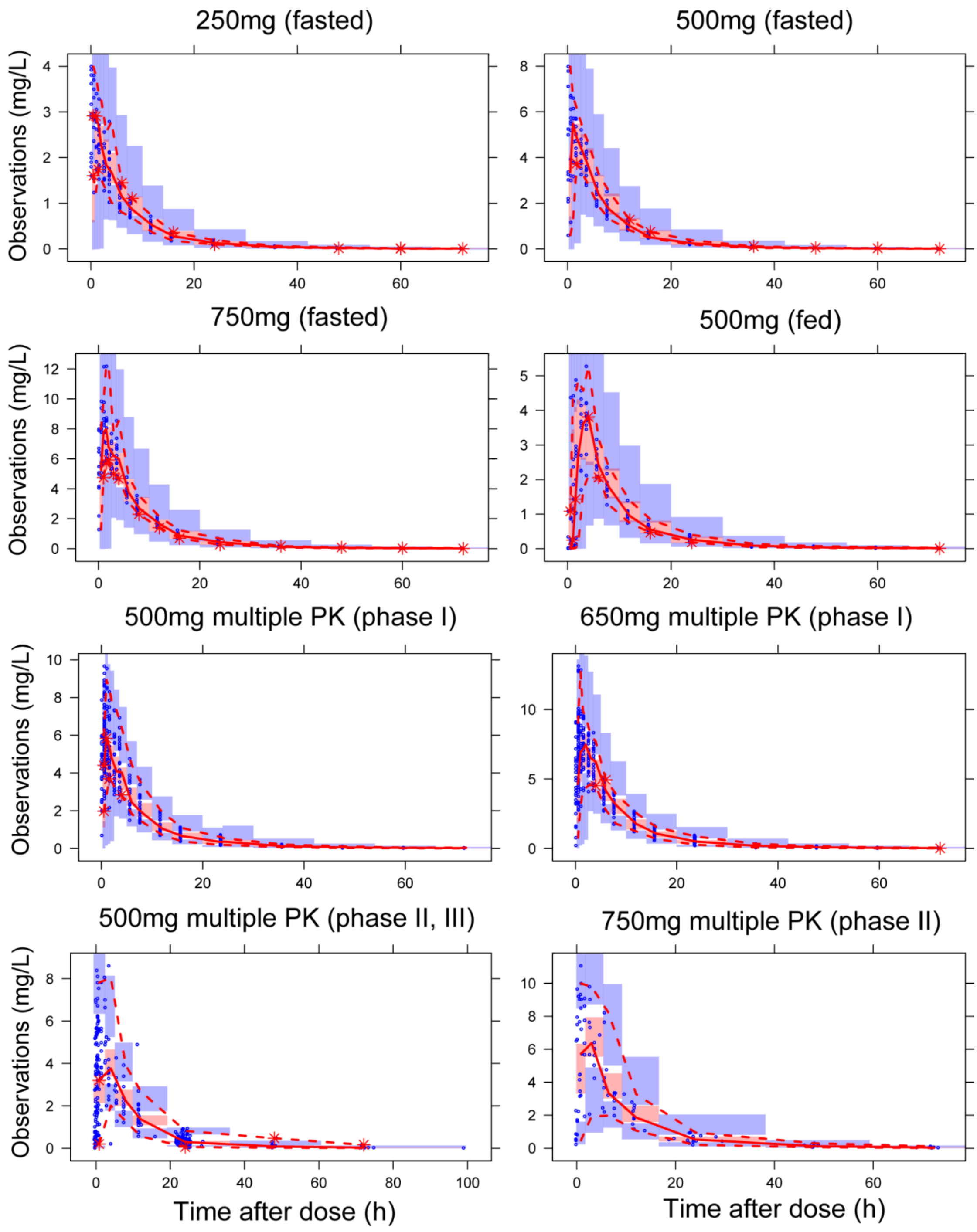


**SUPPLEMENTARY FIGURE 2 |** Visual predictive check for the final population PK model of nemonoxacin. Blue circle: actual value; red solid line: mean of actual value; red dash line: 95% confidence interval for actual value; red area: 95% confidence interval for mean of simulations; blue area: 95% confidence interval for 5% or 95% percentile of simulations. CAP: community-acquired pneumonia; PK: pharmacokinetic.

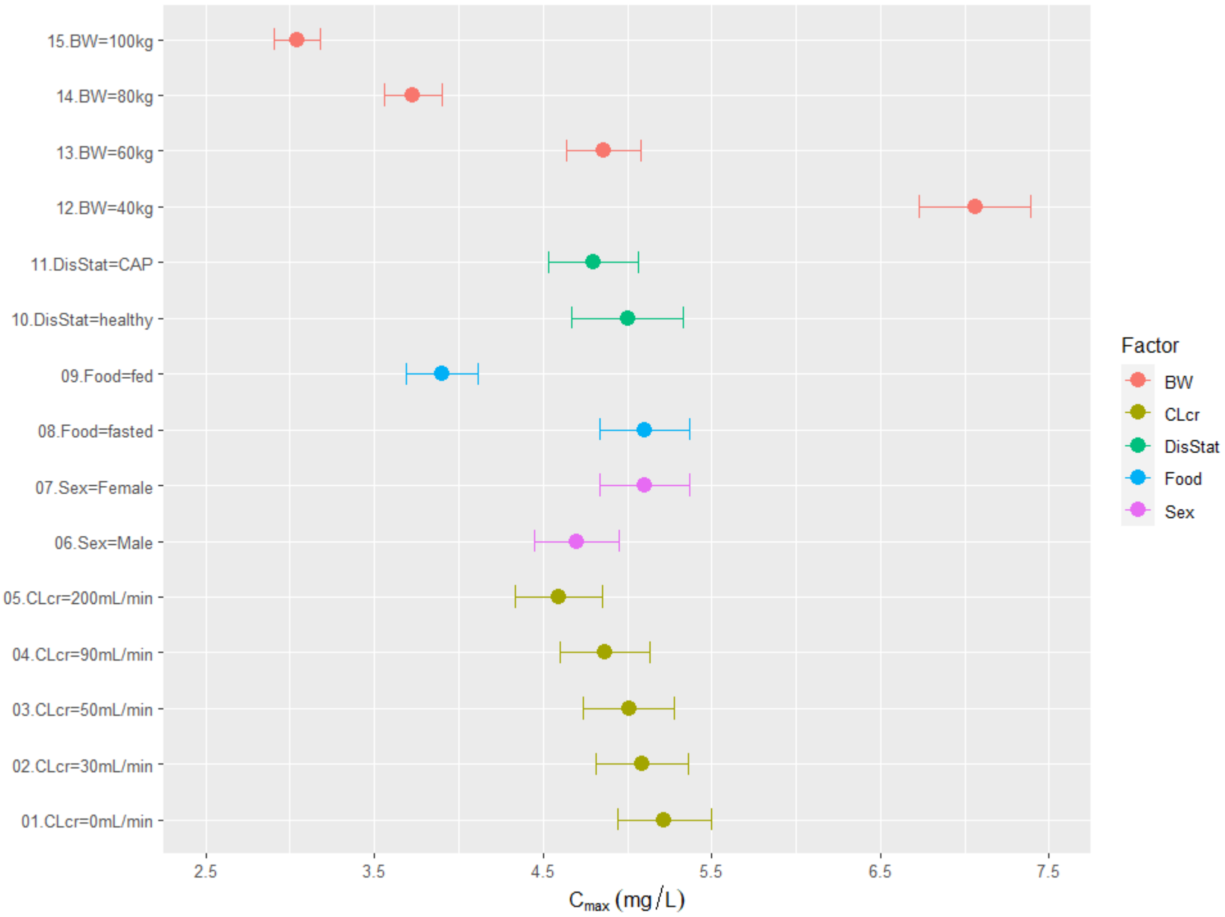


**SUPPLEMENTARY FIGURE 3 |** Effect of covariate on $C_{max}$ of nemonoxacin at steady state. In each line, one covariate had the specified value, and value for other covariates came from the actual. Data was shown as mean±95% confidence interval. Nemonoxacin regimen: oral 500mg every 24h for 10 days. BW: body weight, DisStat: disease status, CAP: community-acquired pneumonia.

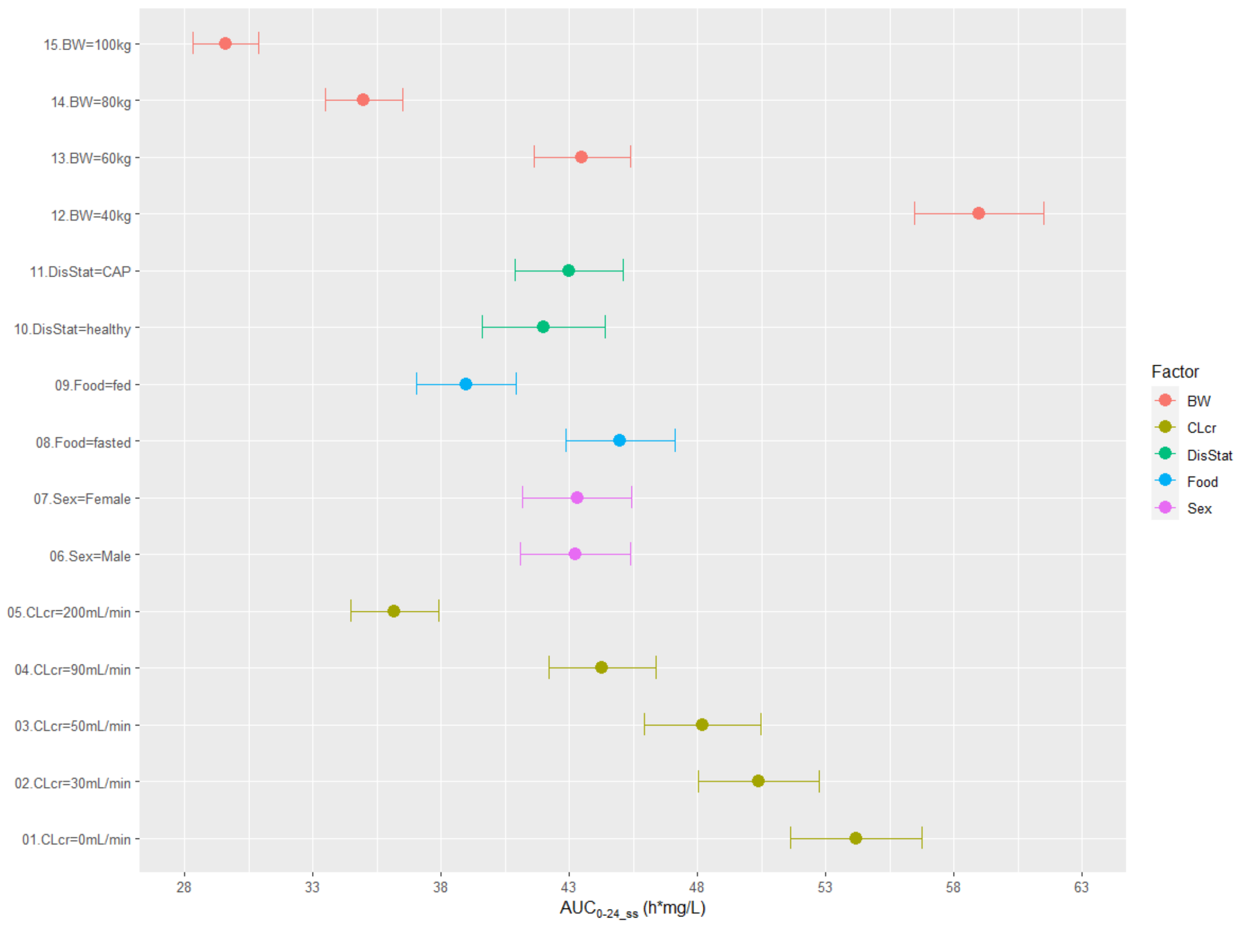


**SUPPLEMENTARY FIGURE 4 |** Effect of covariate on $AUC_{0\text{-}24}$ of nemonoxacin at steady state. In each line, one covariate had the specified value, and value for other covariates came from the actual. Data was shown as mean±95% confidence interval. Nemonoxacin regimen: oral 500mg every 24h for 10 days. BW: body weight, DisStat: disease status, CAP: community-acquired pneumonia.

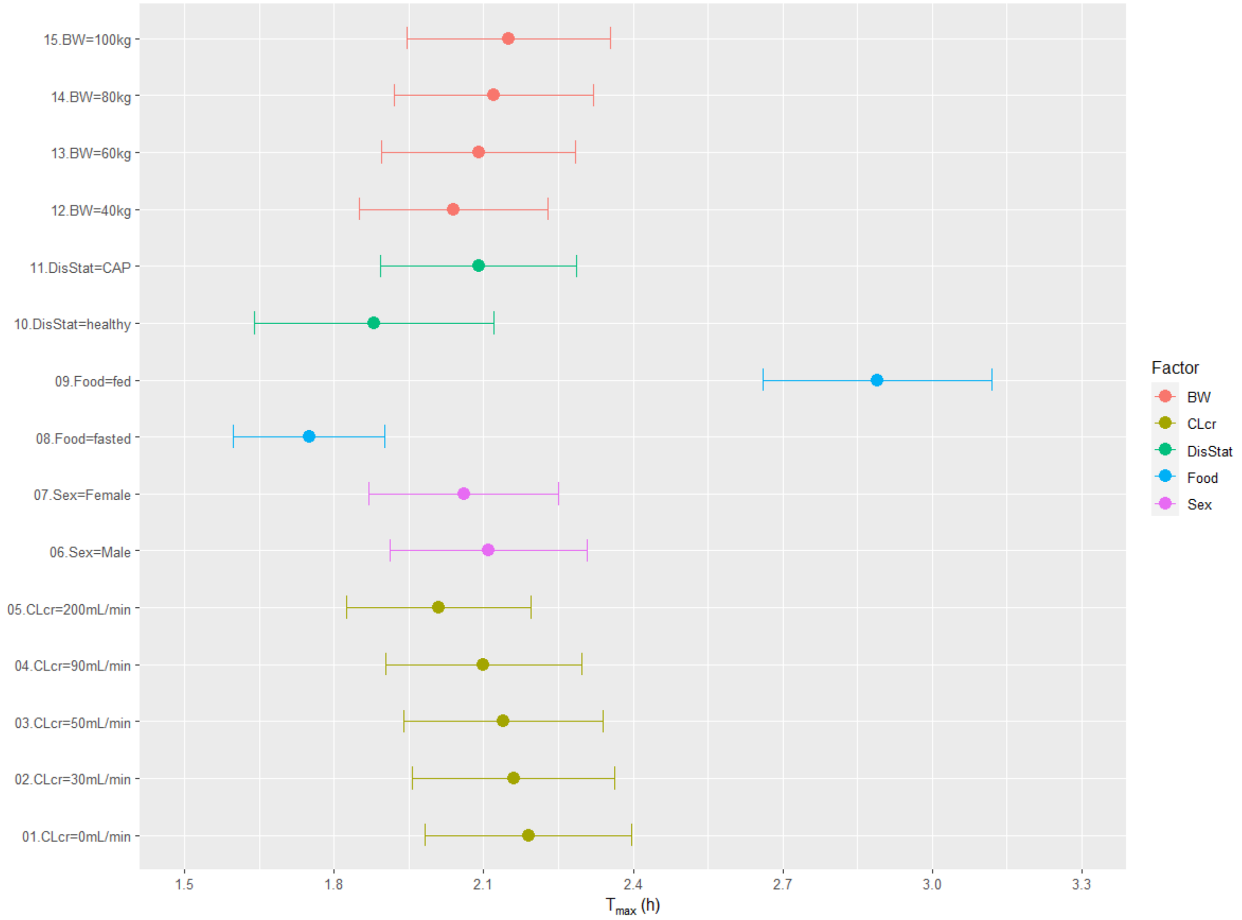


**SUPPLEMENTARY FIGURE 5 |** Effect of covariate on $T_{max}$ of nemonoxacin at steady state. In each line, one covariate had the specified value, and value for other covariates came from the actual. Data was shown as mean ± 95% confidence interval. Nemonoxacin regimen: oral 500mg every 24h for 10 days. BW: body weight, DisStat: disease status, CAP: community-acquired pneumonia.

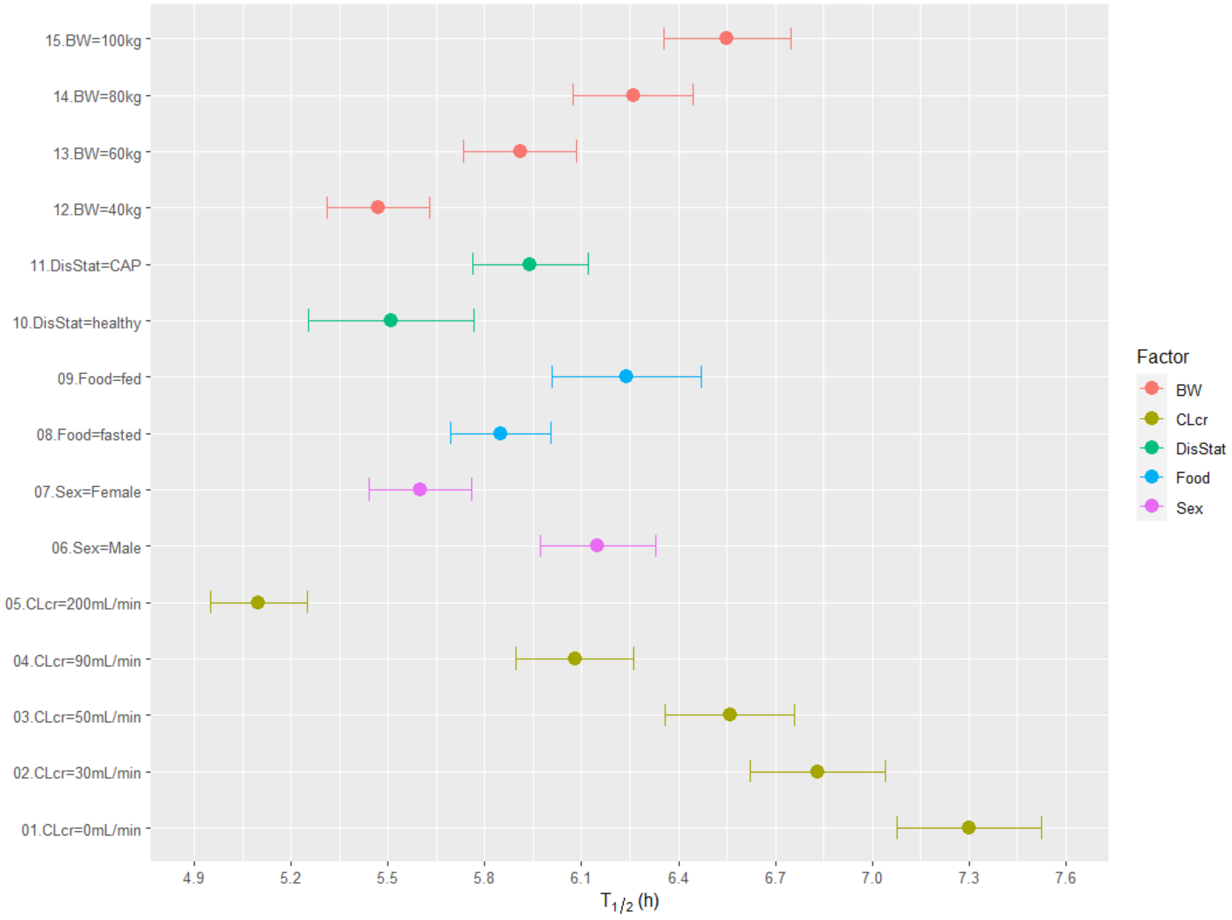


**SUPPLEMENTARY FIGURE 6 |** Effect of covariate on $T_{1/2}$ of nemonoxacin at steady state. In each line, one covariate had the specified value, and value for other covariates came from the actual. Data was shown as mean±95% confidence interval. Nemonoxacin regimen: oral 500mg every 24h for 10 days. BW: body weight, DisStat: disease status, CAP: community-acquired pneumonia.

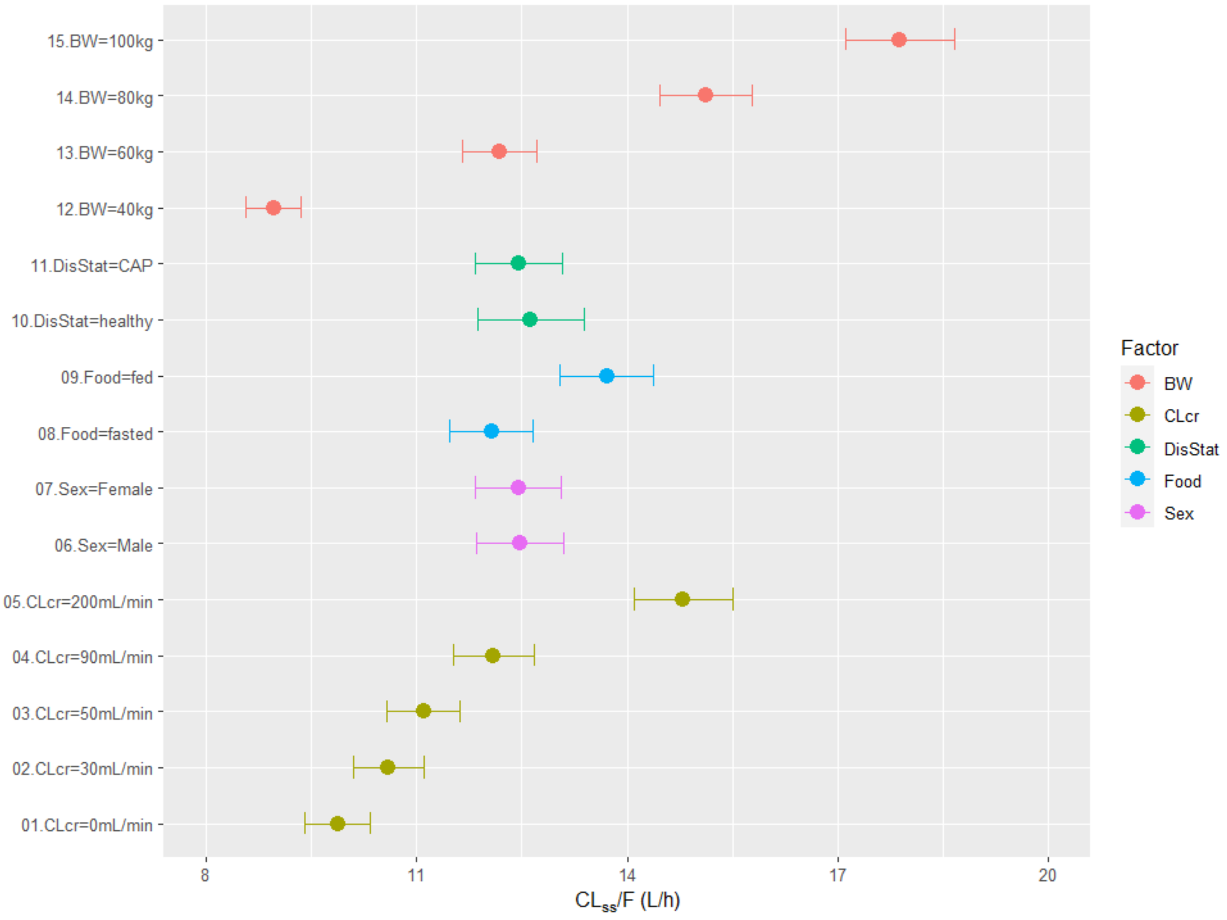


**SUPPLEMENTARY FIGURE 7 |** Effect of covariate on CL/F of nemonoxacin at steady state. In each line, one covariate had the specified value, and value for other covariates came from the actual. Data was shown as mean±95% confidence interval. Nemonoxacin regimen: oral 500mg every 24h for 10 days. BW: body weight, DisStat: disease status, CAP: community-acquired pneumonia.

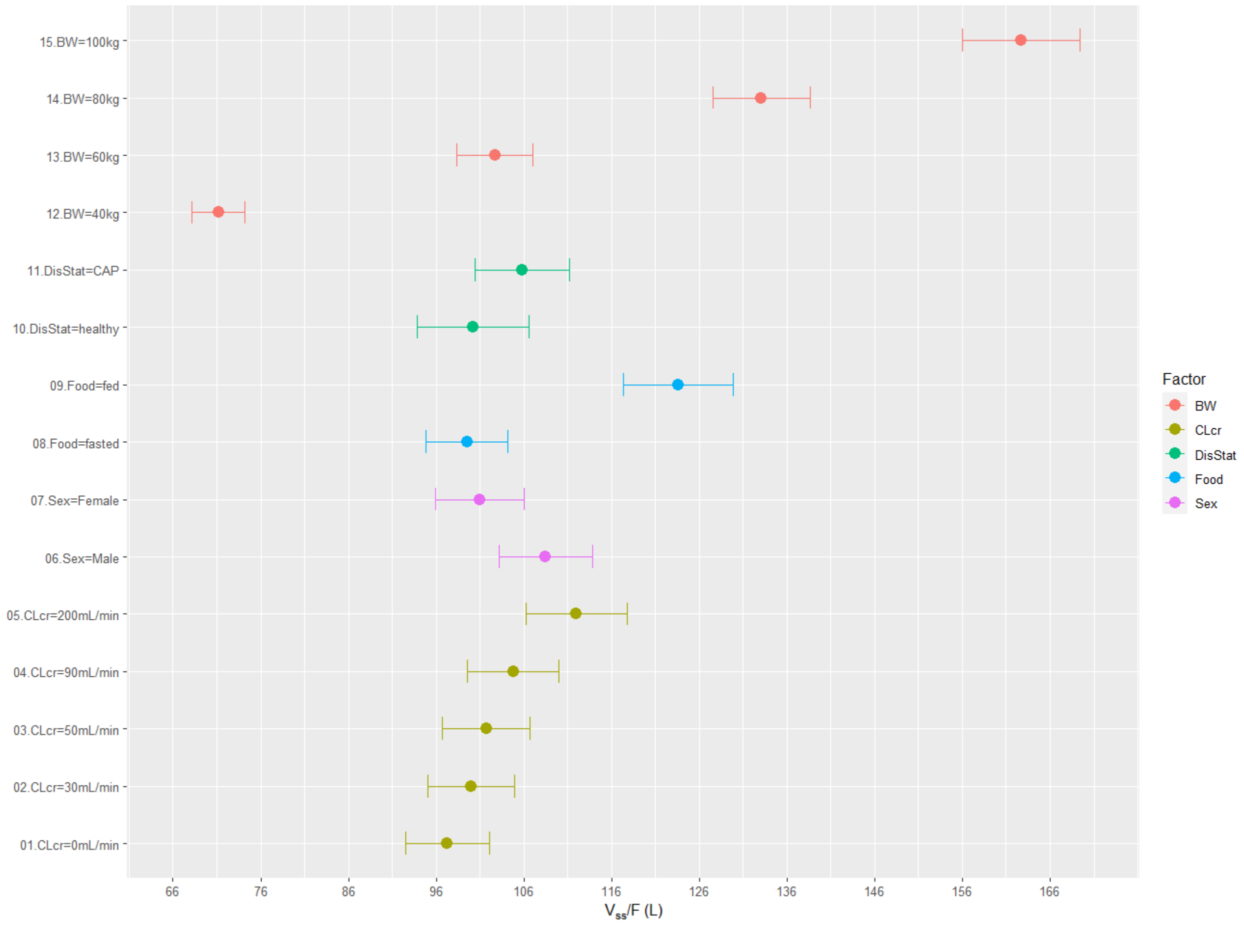


**SUPPLEMENTARY FIGURE 8 |** Effect of covariate on V/F of nemonoxacin at steady state. In each line, one covariate had the specified value, and value for other covariates came from the actual. Data was shown as mean±95% confidence interval. Nemonoxacin regimen: oral 500mg every 24h for 10 days. BW: body weight, DisStat: disease status, CAP: community-acquired pneumonia.

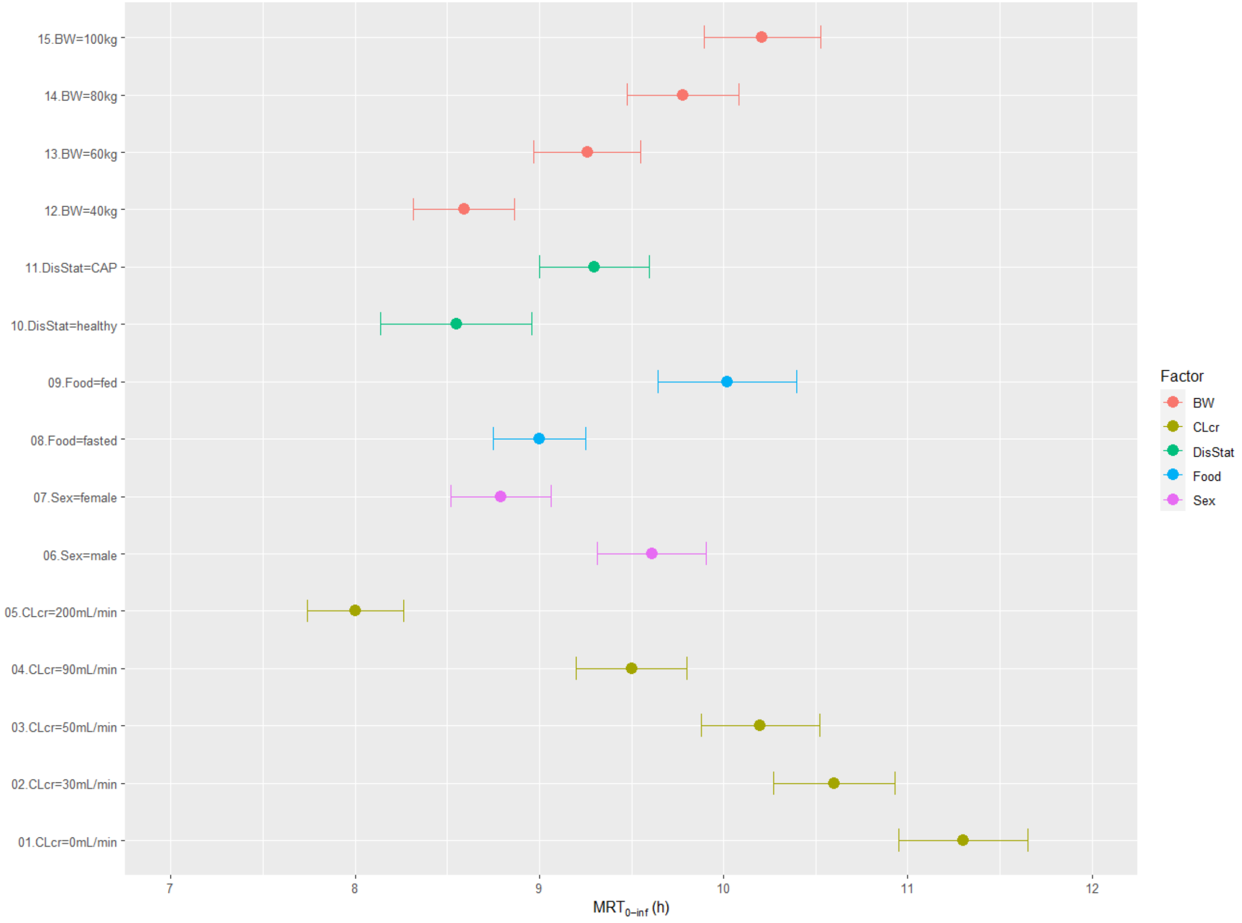


**SUPPLEMENTARY FIGURE 9 |** Effect of covariate on $MRT_{0\text{-inf}}$ of nemonoxacin at steady state. In each line, one covariate had the specified value, and value for other covariates came from the actual. Data was shown as mean±95% confidence interval. Nemonoxacin regimen: oral 500mg every 24h for 10 days. BW: body weight, DisStat: disease status, CAP: community-acquired pneumonia.

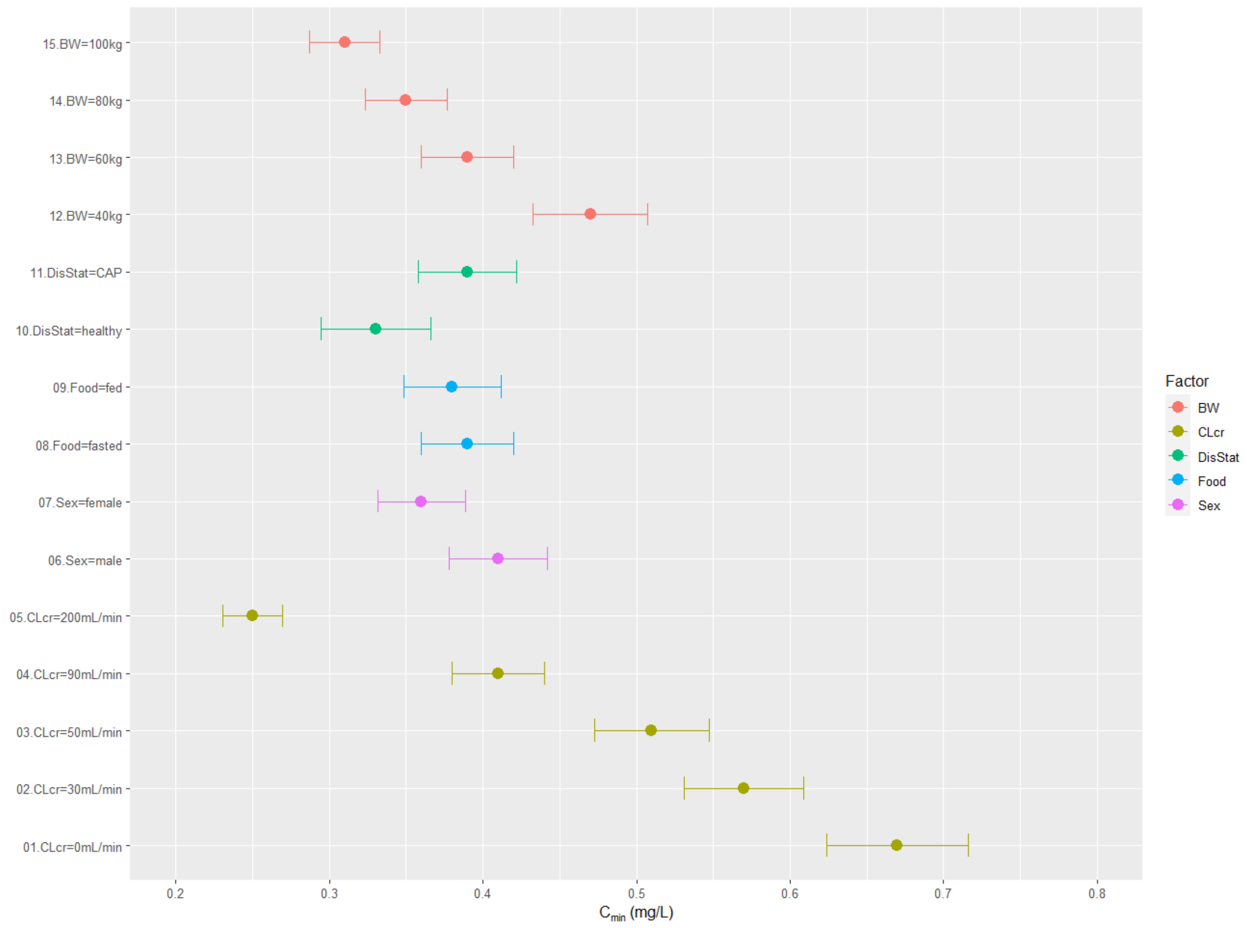


**SUPPLEMENTARY FIGURE 10 |** Effect of covariate on $C_{min}$ of nemonoxacin at steady state. In each line, one covariate had the specified value, and value for other covariates came from the actual. Data was shown as mean±95% confidence interval. Nemonoxacin regimen: oral 500mg every 24h for 10 days. BW: body weight, DisStat: disease status, CAP: community-acquired pneumonia.

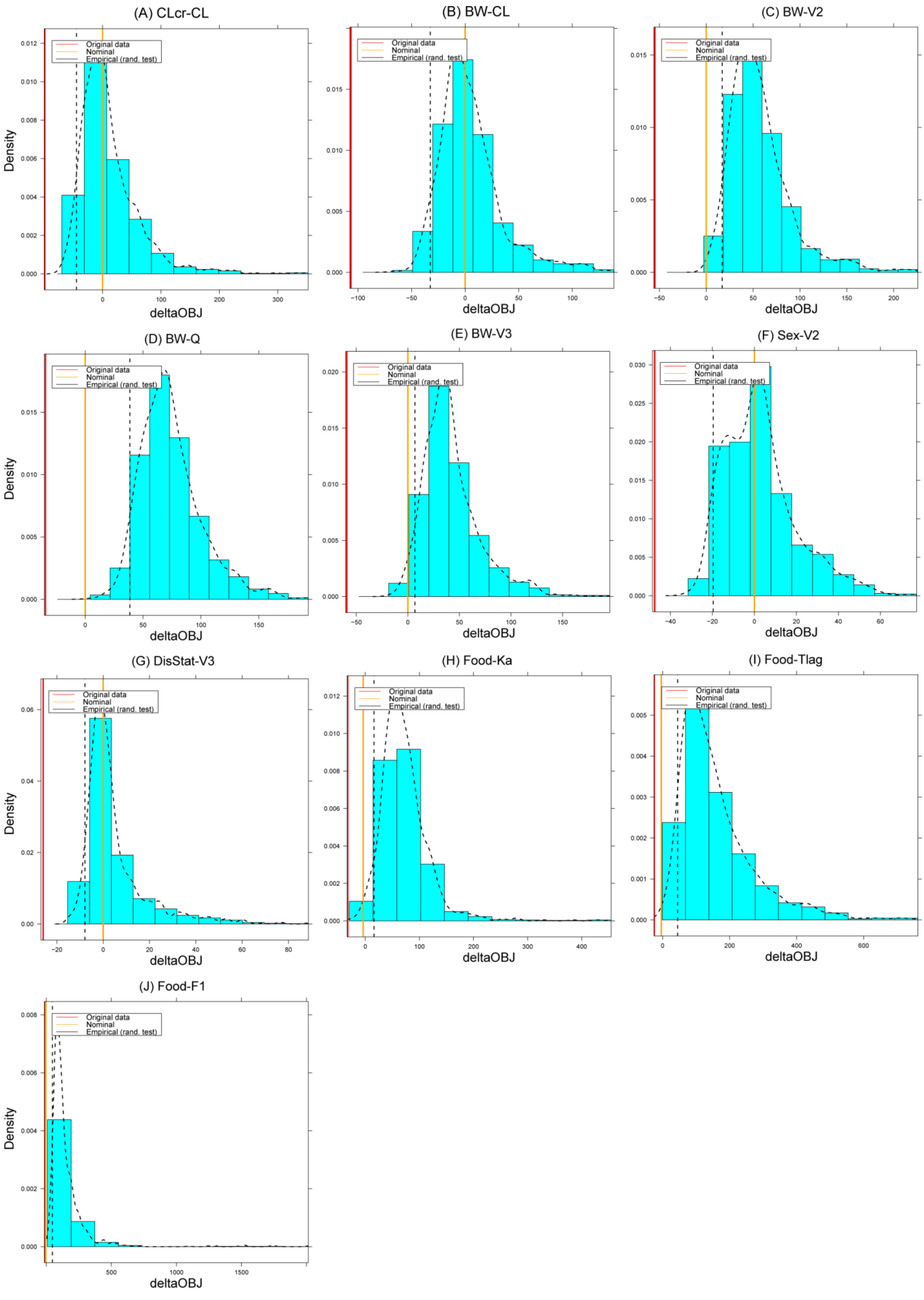
(A) CLcr-CL
(B) BW-CL
(C) BW-V2
(D) BW-Q
(E) BW-V3
(F) Sex-V2
(G) DisStat-V3
(H) Food-Ka
(I) Food-Tlag
(J) Food-F1
Original data
Nominal
Empirical (rand. test)
Density
deltaOBJ

**SUPPLEMENTARY FIGURE 11 |** Change in OBJ (deltaOBJ) for randomization test of final PPK model of nemonoxacin. Name of relation (i.e., covariate-parameter) was shown in the title of each panel. For each relation, the randomization was test 1000 times. During each test, only one covariate (for one parameter) was randomized, other four covariates were included in the model. In the final model, disease status was added on V3; food was added on KA, $T_{lag}$ and F; $CL_{cr}$ was added on CL; sex was added on V2; body weight was added on CL, V2, V3 and Q. deltaOBJ = OBJ (test model) – OBJ (base model). OBJ means objective function value.

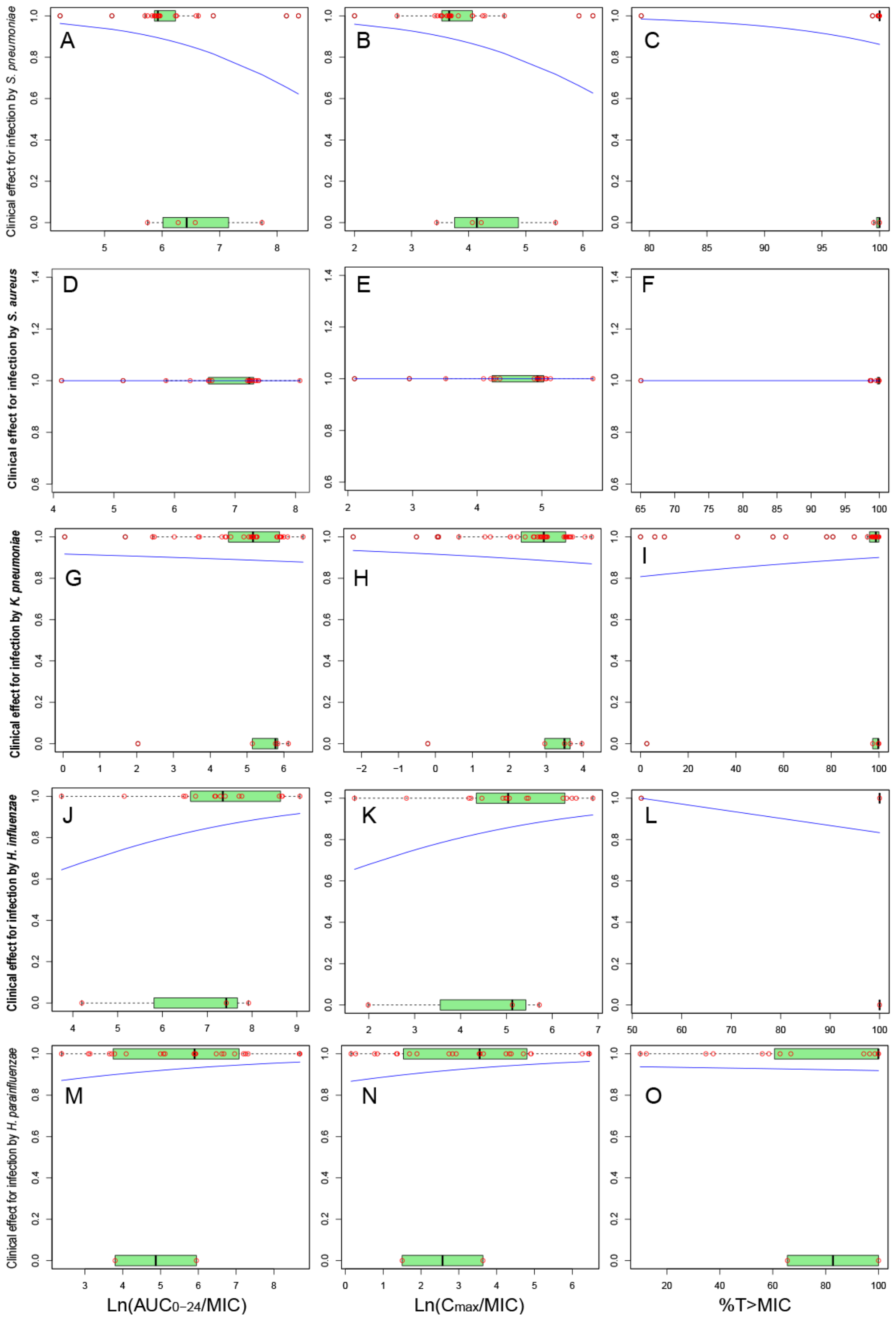


**SUPPLEMENTARY FIGURE 12 |** Correlation between nemonoxacin PK/PD index and clinical efficacy against each bacteria. The left, medium and right columns indicate results for Ln($AUC_{0-24}$/MIC), Ln($C_{max}$/MIC) and %>MIC, respectively. Each row represents results for one bacteria. In each panel, Y axis indicates probability of successful clinical efficacy. Red circle and blue line are actual data and fitting from logistic regression model, respectively. The box plot with light green characterize distribution of actual data: left limit, inner line and right limit of box are 25%, 50% and 75% percentile of actual data, respectively.

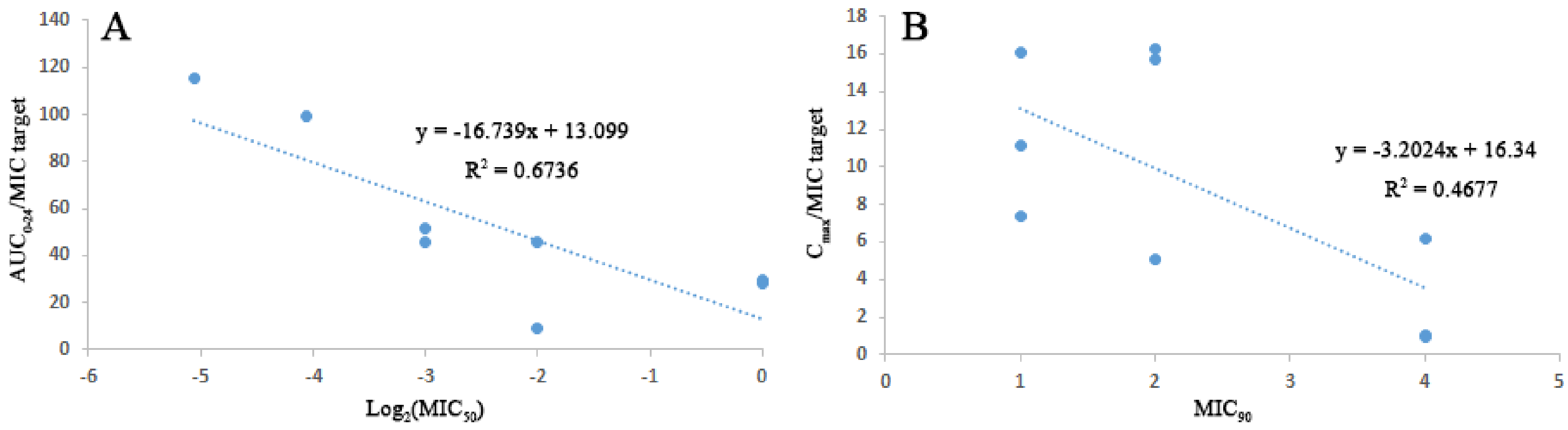


**SUPPLEMENTARY FIGURE 13 |** Correlations between nemonoxacin PK/PD target and MIC against various pathogens. Panel A: $AUC_{0-24}$/MIC target for clinical efficacy; Panel B: $C_{max}$/MIC target for microbiological efficacy. AUC: area under the concentration-time curve; MIC: minimum inhibitory concentration; PK/PD: pharmacokinetic/pharmacodynamic.

**SUPPLEMENTARY TABLE 1 |** Summary of the phase I to III clinical trials of nemonoxacin capsule

| No. | Phase | Design | Number of subjects | Dosing regimen [a] | Sampling time point |
|---|---|---|---|---|---|
| 1 | Phase I<br>Single-dose<br>PK (Stage 1) | Single center, randomized, open-label, single ascending dose PK study | 12 (250 mg)<br>11 (500 mg)[b]<br>12 (750 mg)<br>Healthy subjects | 250 mg<br>500 mg<br>750 mg | 0, 0.5, 1, 1.5, 2, 3, 4, 6, 8, 12,16, 24, 36, 48, 60, and 72 h (dense sampling, 16 points) |
| 2 | Phase I<br>Single-dose<br>PK (Stage 2) | Single center, randomized, open-label study to examine the effect of food on PK | 11 healthy subjects | 500 mg | 0, 0.5, 1, 1.5, 2, 3, 4, 6, 8, 12,16, 24, 36, 48, 60, and 72 h (dense sampling, 16 points) |
| 3 | Phase I<br>Multiple PK | Single center, randomized, open-label study to evaluate tolerability and PK | 12 (500 mg)<br>12 (750 mg)<br>Healthy subjects | 500 mg q24h<br>750 mg q24h<br>10 days | Day 1: 0.5, 1, 1.5, 2, 3, 4, 6, 8, 12, 16, 24 h;<br>Day 3, 5, 8, 9: pre-dose and 1 h post-dose;<br>Day 10: 0, 0.5, 1, 1.5, 2, 3, 4, 6, 8, 12,16, 24, 36, 48, 60, and 72 h<br>36 points for each subject |
| 4 | Phase II<br>Part 1 | Multiple-center, randomized, double-blind, double dummy study to evaluate safety and efficacy | 20 (500 mg)<br>18 (750 mg)<br>CAP patients | 500 mg q24h<br>750 mg q24h<br>7-10 days | (1) Day 4 (0.75, 3, 12 h), Day 7 (48 h post-dose)[c]<br>(2) Day 4 (1.25, 6, 24 h), Day 7 (72 h post-dose)[c]<br>Sparse sampling, 4 points for each patient |
| 5 | Phase II<br>Part 2 | Multiple-center, open-label study to examine the effect of CAP on PK | 12 (500 mg)<br>6 (750 mg)<br>CAP patients | 500 mg q24h<br>750 mg q24h<br>10 days | Day 7 (0.25, 0.5, 1, 2, 4, 8, 12, and 24 h post-dose) + Day 9 (48 h after last dose)<br>9 points for each patient |
| 6 | Phase III | Multiple-center, randomized, double blind, double dummy, parallel study to evaluate treatment efficacy | 69 CAP patients | 500 mg q24h<br>7-10 days | Day 1: 1 ± 0.5 h and 24 ± 2 h after administration<br>Day for last dose: 24 ± 2 h after administration<br>Sparse sampling, 3 points for each patient |

Levofloxacin 500 mg was control treatment group. Abbreviations: CAP: community-acquired pneumonia; PK, pharmacokinetic.

[a] Nemonoxacin was administered orally.

[b] One subject withdrew from this dose level.

[c] Subjects were randomly assigned to one of the two sampling schedules.

Phase I, II and III clinical trial of nemonoxacin capsule were performed during 2008, 2009-2010, 2011-2012, respectively.

**SUPPLEMENTARY TABLE 2 |** Summary of the imputation for the missing covariate data

| Stage | Blood routine | Blood chemistry |
|---|---|---|
| Phase II | None | LDH: 4 subjects (7.1%)<br>$CL^-$, $Na^+$, $K^+$ and CK: 3 subjects (5.4%)<br>TBIL: 2 subjects (3.6%) |
| Phase III | BOS, EOS and MON: 2 subjects (2.9%)<br>WBC, RBC, MCV, PLT, NEU, HGB, HCT, MCH, MCHC and LYM: 1 subject (1.4%) | ALP, RGGT and CK: 4 subjects (5.8%)<br>LDH: 2 subjects (2.9%) |

The missing covariate data were filled with mean value of other subjects. Abbreviations: LDH: lactate dehydrogenase; CK: creatine kinase; TBIL: total bilirubin; BOS: basophil; EOS: eosinophils; MON: monocyte; WBC: white blood cell count; RBC: red blood cell count; MCV: mean corpuscular volume; PLT: platelet; NEU: neutrophil; HGB: hemoglobin; HCT: Hematocrit; MCH: mean corpuscular hemoglobin; MCHC: mean corpuscular hemoglobin concentration; LYM: lymphocyte; ALP: alkaline phosphatase; RGGT: γ-glutamyl transpeptidase

**SUPPLEMENTARY TABLE 3 |** Baseline of the covariates for vital sign, blood routine and biochemistry

| Class | Covariate Name | Unit | Explain | CAP patients (*n*=125) | Healthy subjects (*n*=36) | Change (%) |
|---|---|---|---|---|---|---|
| Vital sign | SBP | mmHg | Systolic blood pressure | 118±11 | 111±10** | 6 |
| | DBP | mmHg | Diastolic blood pressure | 74±7 | 72±7 | 2 |
| | HR | beat/min | Heart rate | 83±13 | 73±9** | 15 |
| | RESPRATE | beat/min | Respiratory rate | 19±1.8 | 19±1.8 | 2 |
| | BODYTEMP | ℃ | Body temperature | 38.1±0.8 | 36.3±0.4** | 5 |
| | QTC | ms | QTc interval | 400±23 | 402±22 | 0 |
| Blood routine | WBC | \ | White blood cell count | 1.27±0.66 | 0.68±0.14** | 87 |
| | RBC | \ | Red blood cell count | 1.01±0.11 | 1.10±0.11** | -8 |
| | PLT | \ | Platelet | 1.11±0.38 | 1.17±0.20 | -5 |
| | NEU | \ | Neutrophils | 1.17±0.19 | 1.03±0.14** | 14 |
| | EOS | \ | Eosinophils | 0.52±0.51 | 0.67±0.54 | -23 |
| Blood biochemistry | ALT | \ | Alanine aminotransferase | 1.15±0.83 | 0.64±0.30** | 79 |
| | AST | \ | Aspartate aminotransferase | 1.30±0.73 | 1.31±0.27 | -1 |
| | TBIL | \ | total bilirubin | 1.04±0.48 | 0.98±0.35 | 6 |
| | ALP | \ | alkaline phosphatase | 0.99±0.42 | 0.90±0.24 | 10 |
| | RGGT | \ | γ-Glutamyltransferase | 1.29±1.36 | 0.48±0.16** | 170 |
| | CPK | \ | Creatine kinase | 1.13±1.08 | 0.86±0.28* | 32 |
| | BUN | \ | Blood urea nitrogen | 0.88±0.28 | 0.95±0.20** | -8 |
| | CCR | \ | Serum creatinine | 0.94±0.24 | 0.68±0.13** | 38 |
| | GLU | \ | Glucose | 1.13±0.32 | 1.04±0.09** | 9 |
| | TPR | \ | Total protein | 1.02±0.10 | 1.03±0.07 | -1 |
| | UA | \ | Uric acid | 1.02±0.30 | 1.08±0.19 | -6 |
| | Chloridion | \ | Chloride ion | 1.00±0.03 | 1.04±0.02** | -4 |
| | Sodium | \ | Sodium ion | 0.99±0.02 | 1.00±0.02 | -1 |
| | Kalium | \ | Potassium ion | 0.94±0.15 | 0.92±0.08 | 2 |
| Other | Center | | Clinical trial center (Huashan hospital/other) | 14/111 | 36/0 | NA |

Note 1: For continuous covariates, results were shown as Mean±SD. For categorical covariates, results were shown as the frequency.

Note 2: Covariates for blood routine and blood biochemistry were shown as the ratio calculated as: measured value/[0.5×(ULN+LLN)]. ULN: upper limit of normal value. LLN: lower limit of normal value

Note 3: Compared to CAP patients: * P<0.05, ** P<0.01 (t test)

Note 4: Change (%) = [Mean (CAP)/Mean (Healthy)-1]×100%

Note 5: NA: not applicable

**SUPPLEMENTARY TABLE 4 |** Correlation of PK parameters for the final PPK model of nemonoxacin ($R^2$)

| | *CL* | *V2* | *Q* | *V3* | *KA* | TLAG | *F* | CL-IIV | V2-IIV | V3-IIV | KA_IIV | CL-IOV1 | CL-IOV2 | TLAG-IIV |
|---|---|---|---|---|---|---|---|---|---|---|---|---|---|---|
| V2 | 0.51 | | | | | | | | | | | | | |
| Q | 0.60 | 0.91 | | | | | | | | | | | | |
| V3 | 0.34 | 0.68 | 0.73 | | | | | | | | | | | |
| KA | 0.01 | 0.00 | 0.00 | 0.05 | | | | | | | | | | |
| TLAG | 0.00 | 0.00 | 0.00 | 0.00 | 0.15 | | | | | | | | | |
| F | 0.01 | 0.00 | 0.00 | 0.03 | 0.20 | 0.08 | | | | | | | | |
| CL-IIV | 0.08 | 0.01 | 0.00 | 0.00 | 0.01 | 0.00 | 0.11 | | | | | | | |
| V2-IIV | 0.07 | 0.00 | 0.01 | 0.00 | 0.16 | 0.02 | 0.02 | 0.55 | | | | | | |
| V3-IIV | 0.00 | 0.01 | 0.00 | 0.04 | 0.00 | 0.02 | 0.07 | 0.11 | 0.01 | | | | | |
| KA_IIV | 0.01 | 0.00 | 0.00 | 0.00 | 0.58 | 0.10 | 0.06 | 0.03 | 0.24 | 0.00 | | | | |
| CL-IOV1 | 0.17 | 0.02 | 0.01 | 0.01 | 0.00 | 0.00 | 0.07 | 0.21 | 0.10 | 0.00 | 0.01 | | | |
| CL-IOV2 | 0.00 | 0.00 | 0.00 | 0.00 | 0.00 | 0.01 | 0.03 | 0.63 | 0.36 | 0.14 | 0.02 | 0.03 | | |
| TLAG-IIV | 0.00 | 0.00 | 0.00 | 0.00 | 0.13 | 0.79 | 0.03 | 0.00 | 0.02 | 0.01 | 0.18 | 0.01 | 0.02 | |
| F-IIV | 0.02 | 0.00 | 0.00 | 0.00 | 0.07 | 0.04 | 0.83 | 0.14 | 0.02 | 0.06 | 0.07 | 0.10 | 0.03 | 0.03 |

Note 1: The cells with $R^2$>0.7 are marked with red background
Note 2: The cells with $R^2$ between 0.3-0.7 are marked with green background
Note 3: For the cells with $R^2$=1 (e.g., CL vs CL), the values are not shown in the table
Note 4: CL_IOV is for occasion 1 (time≤72h), while CL_IOV2 is for occasion 2 (time>72h)
Note 5: IIV: inter-individual variability, IOV: inter-occasion variability

**SUPPLEMENTARY TABLE 5 | Summary of deltaOBJ (dOBJ) in the randomization test**

| Relation name for randomization test | Mean of actual dOBJ | Actual dOBJ at percentile (P=0.05) | Observed dOBJ in the final model |
|---|---|---|---|
| $CL_{cr}$-CL | +11 | -45 | -99 |
| BW-CL | +5 | -33 | -107 |
| BW-V2 | +57 | 0 | -55 |
| BW-Q | +74 | 0 | -34 |
| BW-V3 | +42 | 0 | -59 |
| Sex-V2 | +3 | -20 | -48 |
| DisStat-V3 | +5 | -8 | -26 |
| Food-Ka | +72 | 0 | -33 |
| Food-$T_{lag}$ | +160 | 0 | -25 |
| Food-F1 | +150 | 0 | -14 |

deltaOBJ was obtained according to: OBJ (test model) – OBJ (base model), where base model is reduced model without the relation. Relation was shown as ‘Covariate-Parameter’. For each relation, randomization test was performed 1000 times. DisStat: disease status, $CL_{cr}$: creatinine clearance, BW: body weight, OBJ: objective function value.
Reason for the actual dOBJ at percentile (P=0.05) is 0 was that all dOBJ were positive in the randomization test.

**SUPPLEMENTARY TABLE 6 |** Correlation between nemonoxacin PK/PD index and clinical efficacy for CAP caused by specific pathogen

| Pathogen | N | Ln($AUC_{0\text{-}24}$/MIC) | Ln($C_{max}$/MIC) | %T>MIC | Comment |
|---|---|---|---|---|---|
| *S. pneumoniae* | 31 | Slope = -0.7<br>*P* = 0.27 | Slope = -0.6<br>*P* = 0.28 | Slope = -0.1<br>*P* = 0.77 | Concentration-dependent, PK/PD index maybe AUC/MIC |
| *S. aureus* | 20 | Slope = 6E-7<br>*P* = 1.0 | Slope = 5E-7<br>*P* = 1.0 | Slope = 3E-9<br>*P* = 1.0 | All S. aureus strains were sensitive to nemonoxacin (MIC level was low), all patients were cured, hence the slope was zero |
| *K. pneumoniae* | 45 | Slope = -0.1<br>*P* = 0.86 | Slope = -0.1<br>*P* = 0.77 | Slope = 0.01<br>*P* = 0.59 | Primarily time-dependent. PK/PD index maybe %T>MIC based on postive slope and the lowest *P* value |
| *H. influenzae* | 19 | Slope = 0.3<br>*P* = 0.40 | Slope = 0.3<br>*P* = 0.41 | Slope = -0.3<br>*P* = 1.0 | Concentration-dependent, PK/PD index maybe AUC/MIC |
| *H. parainfluenzae* | 26 | Slope = 0.2<br>*P* = 0.62 | Slope = 0.2<br>*P* = 0.60 | Slope = -3E-3<br>*P* = 0.91 | Concentration-dependent, PK/PD index maybe $C_{max}$/MIC because *P* value is the lowest |

Slope and *P* value were based on logistic regression analysis. P value is for slope, the test statistic is Wald $\chi^2$, the formula is $\chi^2$=(slope/standard error(slope))$^2$. If P value is lower, the significance of the logistic regression model is higher.

**SUPPLEMENTARY TABLE 7 |** PK parameter of nemonoxacin in healthy subjects following single oral administration of 500mg nemonoxacin capsule

| Parameter | $AUC_{0\text{-}inf}$ | $C_{max}$ | $T_{max}$ | $T_{1/2}$ | $V_d/F^{\#}$ | $V_d/F$ | $CL/F^{\#}$ | CL/F | Body weight | Reference |
|---|---|---|---|---|---|---|---|---|---|---|
| Unit | h·mg/L | mg/L | h | h | L/kg | L | L/h/kg | L/h | kg | |
| USA | 32.36 | 3.41 | 2 | 14.75 | 4.25 | 313.65 | 0.2 | 14.76 | 73.8 | Lin, Antimicrob Agents Chemother, 2010 |
| China | 42.4 | 5.91 | 1.14 | 12.8 | 3.8 | 228.38 | 0.2 | 12.02 | 60.1 | Guo, Clin Drug Investig, 2012 |
| Bias | 10.04 | 2.5 | -0.86 | -1.95 | -0.45 | -85.27 | 0 | -2.74 | -13.7 | |
| Bias (%) | 31 | 73 | -43 | -13 | -11 | -27 | 0 | -19 | -19 | |

Note 1: Bias = (Para_China - Para_USA)*100%. Bias(%) = Bias/Para_USA * 100%. 'Para' means PK parameter
Note 2: # means correction by body weight

**SUPPLEMENTARY TABLE 8 |** PK parameter of nemonoxacin at steady state in healthy subjects following multiple oral administration of 500mg nemonoxacin capsule

| Parameter | $AUC_{0-24,ss}$ | $C_{max,ss}$ | $T_{max}$ | $T_{1/2}$ | $CL_{ss}/F$ | $V_{ss}/F$ | Body weight | Reference |
|---|---|---|---|---|---|---|---|---|
| Unit | h·mg/L | mg/L | h | h | L/h | L | kg | |
| USA | 38.6 | 5.6 | 1.31 | 18.6 | 11 | - | 80.04 | Chung DT, Antimicrob Agents Chemother, 2010 |
| China | 46.9 | 7.02 | 1.25 | 12.6 | 11.362 | 112.5 | 59.8 | Guo, Clin Drug Investig, 2012 |
| Bias | 8.3 | 1.42 | -0.06 | -6 | 0.362 | \ | -20.24 | |
| Bias (%) | 22 | 25 | -5 | -32 | 3 | \ | -25 | |

Note 1: Bias = (Para_China - Para_USA)*100%. Bias(%) = Bias/Para_USA * 100%. ‘Para’ means PK parameter
Note 2: ‘-’ means not reported, ‘\’ means not calculate

**SUPPLEMENTARY TABLE 9 |** Probability for ratio of $AUC_{0\text{-}inf}$ of nemonoxacin greater than 2 in CAP patients with $CL_{cr}$=0 mL/min

| Control | Level of $CL_{cr}$ (mL/min) | | | | |
|---|---|---|---|---|---|
| | 300 | 250 | 200 | 150 | 90 |
| Probability (%) | 48% | 40% | 31% | 23% | 13% |

Note: ratio of $AUC_{0\text{-}inf} = AUC_{0\text{-}inf\_CLcr=0}/AUC_{0\text{-}inf\_control}$. Nemonoxacin regimen: 500mg (q24h) for 10 days. The $AUC_{0\text{-}inf}$ was calculated based on 0-72h simulation data after last dose.

**Acknowledgement**

We would like to express our gratitude to the following 69 hospitals for their contribution to this PPK study of nemonoxacin by actively participating in phase II/III clinical trials: Huashan Hospital, Fudan University; Shengjing Hospital, China Medical University; Putuo Hospital Affiliated to Shanghai University of Traditional Chinese Medicine; The First Affiliated Hospital, Zhejiang University; Central Hospital of Changsha; East Hospital, Tongji University; Gansu Provincial People's Hospital; Jiangxi Provincial People's Hospital; West China Hospital, Sichuan University; The First Affiliated Hospital, Shanxi Medical University; The Second Affiliated Hospital, Wenzhou Medical University; Hainan Provincial People's Hospital; Wuhan General Hospital of Guangzhou Command; People's Hospital of Shenzhen; Affiliated Hospital of Hainan Medical University; The Second Artillery General Hospital; Hunan Provincial People's Hospital; Beijing Anzhen Hospital, Capital Medical University; Nanjing General Hospital; Taihe Hospital, Hubei University of Medicine; the Second Affiliated Hospital, Nanchang University; Changzheng Hospital; Daping Hospital, the Third Military Medical University; the Second Affiliated Hospital, Zhongshan University; the first Affiliated Hospital, Fujian Medical University; the First Affiliated Hospital of Chongqing Medical University; Guangzhou Red Cross Hospital; Huadong Hospital, Fudan University; the Second Affiliated Hospital, Lanzhou University; the First Affiliated Hospital, Xinjiang Medical University; Shanghai Jiao Tong University Affiliated Sixth People's Hospital; Peking Union Medical College Hospital; People's Hospital of Wuhan University; Chi Mei Medical Center; Tri-Service General Hospital; Chung Shan Medical University Hospital; Taichung Veterans General Hospital; Kaohsiung Chang Gung Memorial Hospital; China Medical University Hospital, Taipei; Cheng Hsin General Hospital; EDa Hospital, Kaohsiung City; National Taiwan University Hospital; Ditmanson Medical Foundation Chia-Yi Christian Hospital; Taipei Veterans General Hospital; Second Affiliated Hospital of Dalian Medical University; The Third Xiangya Hospital of Central South University; Xiangya Hospital Central South University; Peking University Third Hospital; Peking University People's Hospital; Institute of Clinical Pharmacology, Peking University; Far Eastern Memorial Hospital; Second Affiliated Hospital of Xi'an Jiaotong University; The First Affiliated Hospital of the Third Military Medical University; Shenyang Military Region General Hospital; Yuan's General Hospital; Kunming Military Region General Hospital; Hangzhou First People's Hospital; Affiliated Hospital of Qingdao University; Huaian First Hospital Affiliated to Nanjing Medical University; Jinan Central Hospital; Affiliated Beijing Chaoyang Hospital of Capital Medical University; Affiliated Hospital of Guilin Medical College; Kaohsiung Medical University Chung-Ho Memorial Hospital; The Second Affiliated Hospital of the Third Military Medical University; Shuang-Ho Hospital; and Cheng Ching Hospital.
